# NAT10: An RNA cytidine transferase regulates fatty acid metabolism in cancer cells

**DOI:** 10.1002/ctm2.1045

**Published:** 2022-09-23

**Authors:** Mahmood Hassan Dalhat, Mohammed Razeeth Shait Mohammed, Hind Ali Alkhatabi, Mohd Rehan, Aamir Ahmad, Hani Choudhry, Mohammad Imran Khan

**Affiliations:** ^1^ Department of Biochemistry King Abdulaziz University Jeddah Saudi Arabia; ^2^ Centre for Artificial Intelligence in Precision Medicines King Abdulaziz University Jeddah Saudi Arabia; ^3^ Department of Biochemistry College of Science University of Jeddah Jeddah Saudi Arabia; ^4^ King Fahd Medical Research Centre King Abdulaziz University Jeddah Saudi Arabia; ^5^ Department of Medical Laboratory Technology, Faculty of Applied Medical Sciences King Abdulaziz University Jeddah Saudi Arabia; ^6^ Translational Research Institute Hamad Medical Corporation Doha Qatar

**Keywords:** ac4C, cancer, fatty acid metabolism, NAT10

## Abstract

**Background:**

*N*‐4 cytidine acetylation (ac4C) is an epitranscriptomics modification catalyzed by *N*‐acetyltransferase 10 (NAT10); important for cellular mRNA stability, rRNA biogenesis, cell proliferation and epithelial to mesenchymal transition (EMT). However, whether other crucial pathways are regulated by NAT10‐dependent ac4C modification in cancer cells remains unclear. Therefore, in this study, we explored the impact of NAT10 depletion in cancer cells using unbiased RNA‐seq.

**Methods:**

High‐throughput sequencing of knockdown NAT10 in cancer cells was conducted to identify enriched pathways. Acetylated RNA immunoprecipitation‐seq (acRIP‐seq) and RIP‐PCR were used to map and determine ac4C levels of RNA. Exogenous palmitate uptake assay was conducted to assess NAT10 knockdown cancer cells using Oil Red O staining and lipid content analysis. Gas‐chromatography–tandem mass spectroscopy (GC/MS) was used to perform untargeted lipidomics.

**Results:**

High‐throughput sequencing of NAT10 knockdown in cancer cells revealed fatty acid (FA) metabolism as the top enriched pathway through the Kyoto Encyclopedia of Genes and Genomes (KEGG) pathway analysis in differentially downregulated genes. FA metabolic genes such as *ELOLV6, ACSL1*, *ACSL3*, *ACSL4*, *ACADSB* and *ACAT1* were shown to be stabilised via NAT10‐dependent ac4C RNA acetylation. Additionally, NAT10 depletion was shown to significantly reduce the levels of overall lipid content, triglycerides and total cholesterol. Further, NAT10 depletion in palmitate‐loaded cancer cells showed decrease in ac4C levels across the RNA transcripts of FA metabolic genes. In untargeted lipidomics, 496 out of 2 279 lipids were statistically significant in NAT10 depleted cancer cells, of which pathways associated with FA metabolism are the most enriched.

**Conclusions:**

Conclusively, our results provide novel insights into the impact of NAT10‐mediated ac4C modification as a crucial regulatory factor during FA metabolism and showed the benefit of targeting NAT10 for cancer treatment.

## Graphical abstract

- NAT10 is highly expressed in approximately 92% of cancer and it is related to cancer cell survival.
- NAT10 regulates fatty acid metabolism through ac4C‐mediated mRNA stability of ELOVL6, ACSL1, ACSL3, ACSL4, ACADSB and ACAT1.
- Targeting NAT10 could be beneficial for treating severe cancer cases driven by fatty acid metabolism.

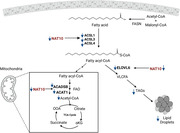

## 1 INTRODUCTION

RNA cytidine acetylation is an important epitranscriptomic event catalyzed by *N*‐acetyltransferase 10 (NAT10) at the N4 position referred to N4‐acetylcytidine (ac4C) modification.^1^, ^2^, ^3^ Studies related to NAT10 mediated ac4C modification have shown its biological impact in many cellular events such as ribosome biogenesis, translational efficiency, bone remodelling, cell proliferation and epithelial to mesenchymal transition (EMT).^1^, ^4^, ^5^, ^6^, ^7^, ^8^, ^9^, ^10^

N4‐cytidine acetylation at position 1 773 (ac4C1773) of 18S rRNA is shown to be essential for 18S rRNA related RNA processing and ribosome biogenesis in saccharomyces cerevisiae.^4^ Another study in human reported acetylation at position 1 842 (ac4C1842) evidently showing the impact of ac4C modification in 18S rRNA biogenesis.^5^ Similarly, the presence of ac4C at anticodon position 34 (ac4C34) of bacterial long or tRNA^Met^ is important for translation fidelity and prevents misdecoding during protein synthesis in *E. coli*.^6^ Additionally, cytidine acetylation promotes translational efficiency through NAT10 mediated deposition of ac4C in the coding sequence (CDS) region of *POLR2A* mRNA transcript in human cervical cancer (HeLa cells).^1^ Notably, N4‐cytidine acetylation at 18S rRNA and tRNA^Ser/Leu^ were also reported in HeLa cells.^1^ Therefore, acetylation of the three major RNAs; rRNA, tRNA, and mRNA provide solid evidence of the impact of RNA acetylation in promoting RNA biogenesis and translational efficiency.

NAT10 regulates osteogenic differentiation of bone marrow derived stem cells (BMSCs) through acetylation of *RUNX2* mRNA transcript. RUNX2 is a critical player in osteoblast differentiation leading to bone formation.^7^ Similarly, NAT10 regulates osteogenic differentiation of mesenchymal stem cells (MSCs) via Gremlin 1 ac4C mediated decay. Gremlin 1 is an antigonist of bone morphogenic protein 2 (BMP2) and BMP4 which play essential role in the bone development.^8^

NAT10 promotes cell proliferation by acetylation *CEP170* mRNA transcript in multiple myeloma. The stability of acetylated CEP170 transcript is associated with cell proliferation and chromosomal instability (CIN).^9^ In gastric cancer, NAT10 was reported to control COL5A1 via NAT10 mediated ac4C pathway, which implicates in EMT and metastasis processes. The ac4C modification is highly enriched in the 3′ UTR on the *COL5A1* mRNA transcript. COL5A1 is positively correlated with VIM and MMP2 known to be critical players of EMT and metastasis.^10^

In bladder cancer, NAT10‐mediated reduction of ac4C modification was reported to suppress the stability and translational efficiency of BCL9L, SOX4 and AKT1, thereby inhibiting cancer progression.^11^

Although many information point to the impact of NAT10‐dependent ac4C modification on different cancer mechanisms, knowledge of the impact of NAT10 in pathways is still obscure. Recently, we reported the effect of Remodelin, the only known small molecule inhibitor of NAT10 on mitochondrial lipid metabolism ^12^. With this information, we decided to explore and identify pathways that are regulated through NAT10‐dependent ac4C RNA acetylation.

## 2 RESULTS

### 2.1 Loss of NAT10 leads to decrease in cell proliferation and cell survival

**FIGURE 1:**
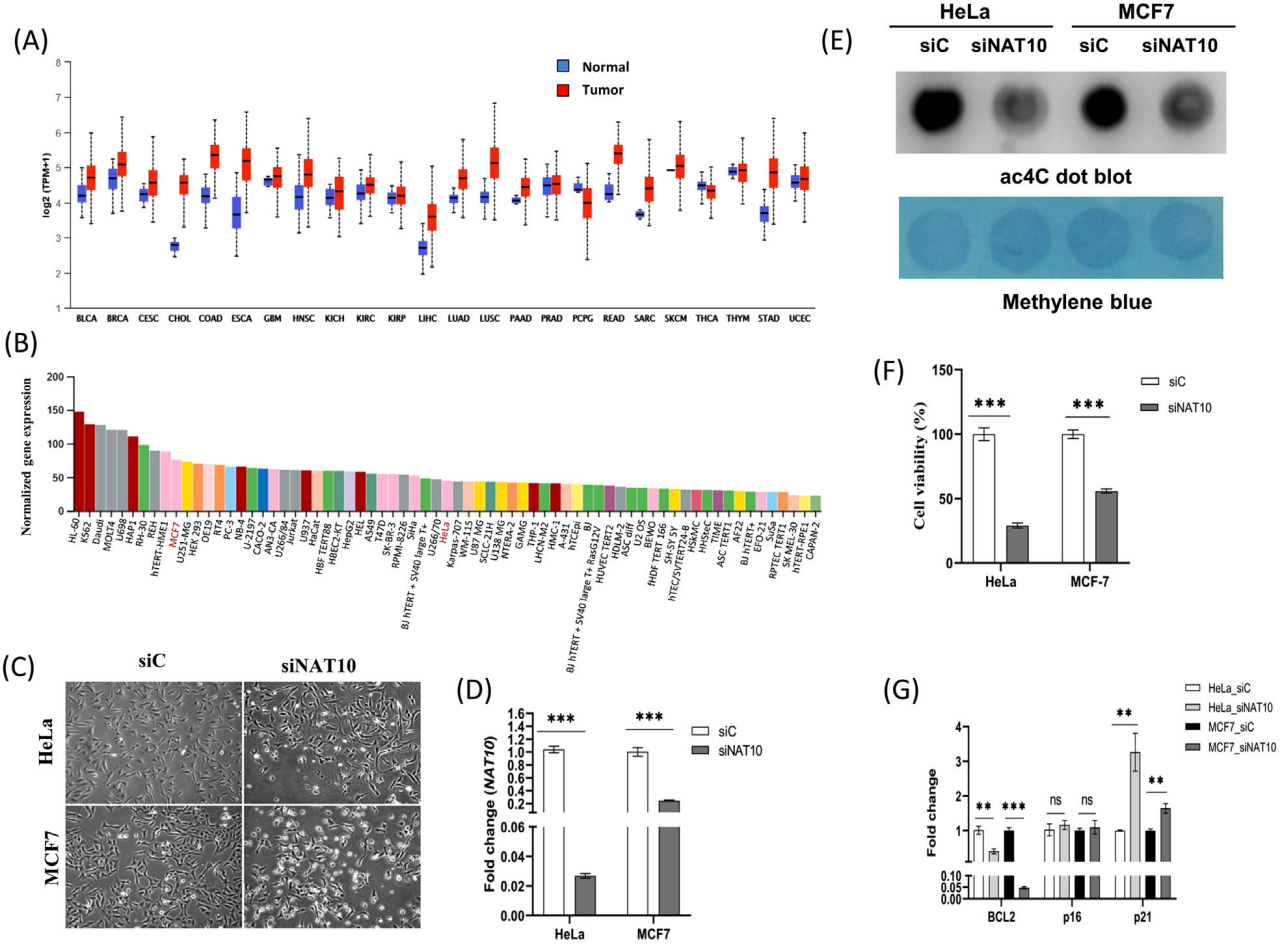
Impact of NAT10 expression in cancer survival. (A) NAT10 expression across different cancer types in TCGA datasets. Bars in blue represent normal tissues and red bars represent tumor tissues. (B) NAT10 RNA expression in HeLa and MCF7 from the human protein atlas. (C) Morphology of HeLa and MCF7 after 24 h transfection with NAT10 siRNA using Nikon microscope at X10 magnification. (D) NAT10 expression levels expression in HeLa and MCF7 after 24 h transfection with NAT10 siRNA. (E) Representation anti‐ac4C dot blot performed on total RNA with methylene as loading control. (F) Cell viability of HeLa and MCF7. Each bar represents cell viability levels after 24 h transfection with NAT10 siRNA. (G) Cell survival genes expression post NAT10 knockdown. All data are represented as mean ± SEM (*n* = 3). **p < .01; ***p < .001; and ns > .05.

To determine the NAT10 expression in cancer and normal tissues, the mRNA data from GTEx and TCGA were retrieved. Interestingly, NAT10 is overexpressed in 92% of cancer compared with normal tissues (Figure 1A). To explore the expression of NAT10 in cell lines, we checked the human protein atlas; among the most highly expressed cell line in solid tumours are HeLa and MCF7, which are cell lines of cervical cancer and breast cancer, respectively (Figure 1B). It is important to note that HeLa was used in an earlier study that showed the role of NAT10 as an RNA cytidine transferase in mRNA and promotes translation efficiency. Therefore, we considered MCF7 and HeLa cells as good candidates for the study of NAT10.

To study the impact of NAT10 on cancer cells, we knockdown both HeLa and MCF7 using NAT10 siRNA, which was confirmed using qRT‐PCR in transfected control (siC) versus NAT10 knockdown cells (siNAT10) (Figure 1C,D). Knockdown of NAT10 was also performed and confirmed in other breast cancer cell lines, that is, MDA‐MB‐231, MDA‐MB‐468, and T47D (Figure S1A). Due to the decreased cell proliferation and cell dead post knockdown with NAT10 siRNA as observed from the cell morphology, we then decided to perform cell viability assay. Notably, the overall ac4C levels of the cancer cells were significantly decreased in NAT10‐deficient cancer cells post transfected with NAT10 siRNA, suggesting that the NAT10‐deficient cancer cells have reduced ac4C levels as well as global acetylated RNAs due to NAT10‐mediated ac4C reduction (Figure 1E). Next, both knockdown cancer cells were subject to cell viability test using 3‐(4,5‐Dimethylthiazol‐2‐yl)‐2,5‐Diphenyltetrazolium Bromide (MTT) assay, and interestingly both cells showed more than 50% reduction in cells viability (Figure 1F).

Additionally, apoptosis assay showed approximately increase in cell death by 16% and 30% for HeLa and MCF7 NAT10 knockdown cells, respectively (Figure S1B). Interestingly, cycle arrest was observed at G2/M phase in both HeLa and MCF7, similar to a previous study (Figure S1C).^1^ To confirm the reduced proliferative and survival effect in NAT10‐deficient cancer cells, we performed expression analysis of some cell proliferation genes such as *BCL2*, *p16*, and *p21*. The expression results from the cell proliferation genes supported the information that NAT10 is crucial for cancer cell proliferation and cell survival (Figure 1G). Previous results have shown that NAT10 deficiency in cancer cells causes decreased cell proliferation and cell survival. We performed mitochondrial membrane potential and dead cell assays to check if the NAT10 defect could also affect mitochondrial biogenesis. Surprisingly, a significant reduction was observed in both cell lines for both experiments suggesting that NAT10 is critical in preserving mitochondrial integrity and preventing mitochondrial‐related cell dead (Figure S1E,F).

Since NAT10 plays a significant role in cancer cell survival and viability, we then performed high‐throughput RNA sequencing to explore possible pathways associated with NAT10.

### 2.2 Fatty acid metabolic pathway is enriched in NAT10 depleted cancer cells

**FIGURE 2:**
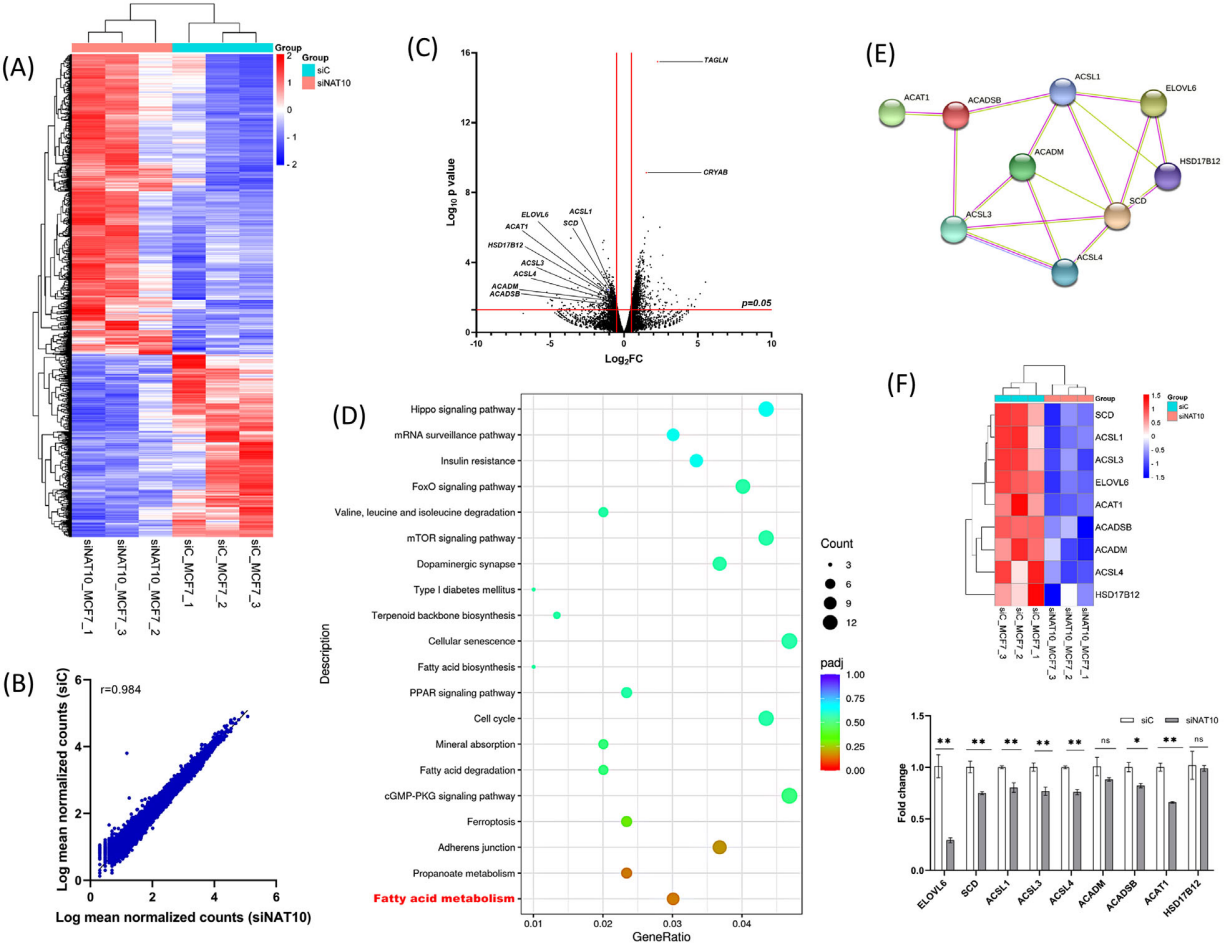
Transcriptome‐wide studies of NAT10 Knockdown MCF7 cells. (A) Heatmap showing expression pattern of genes in MCF7 post transfected with NAT10 siRNA. (B) Scattered plot of deferentially genes of siNAT10 vs. siC in RNA‐seq. (C) Volcano plot showing positions of differentially upregulated and downregulated genes in MCF7 post transfected with NAT10 siRNA. (D) Bubble plot demonstrating the KEGG pathway analysis of MCF7 post transfected with NAT10 siRNA. (E) Network analysis of fatty acid metabolic genes. (F) Validation of the expression levels of fatty acid metabolic genes with RT‐PCR. Bars are represented as mean ± SEM (*n* = 3). *p < .05; **p < .01; ***p < .001; and ns > .05.

Previously, RNA‐seq and acRIP‐seq were performed on HeLa knockout cells; however, the pathways of NAT10 defect were not adequately studied, especially NAT10‐mediated ac4C‐mediated pathways (Figure S2A,B).^1^ Since based on the human protein atlas (https://www.proteinatlas.org/) MCF7 expresses more NAT10 transcripts than HeLa; we decided to perform RNA‐seq in MCF7 depleted NAT10. Triplicate samples from both NAT10 knockdown (siNAT10) and control (siC) were used for RNA‐seq and showed interesting expression patterns when comparing the two conditions (Figure 2A). Interestingly, the scattered plot between siNAT10 versus siC showed a significant correlation at 0.98 (Figure 2B). We identified 2 155 differentially expressed genes (DEGs) that were significant in high‐throughput RNA sequencing of MCF7 siNAT10. The DEGs were stratified into differentially expressed upregulated genes (DUGs) and differentially expressed downregulated genes (DDGs) (Figure 2C). These genes were then subjected to gene ontologies and Kyoto Encyclopedia of Genes and Genomes (KEGG) pathways analysis. Since we were interested in studying pathways related to NAT10 deficiency, we focused on the DDGs. The top biological processes identified were regulation of the muscle tissue development, regulation of anatomic structure size, regulation of striated muscle development, regulation of muscle organ development, and regulation of cellular component size (Figure S3A). The top enriched pathways from KEGG analysis were fatty acid (FA) metabolism, propanoate metabolism, adherens junction, ferroptosis and cGMP‐PKG signalling pathway (Figure 2D). Based on the KEGG analysis, we further explore the FA metabolic pathway (Table S1). The genes identified under the FA metabolic pathway include the following: Acyl‐CoA synthetase long chain‐1 (*ACSL1*), *ACSL3*, *ACSL4*, Elongation of very long chain FAs protein 6 (*ELOVL6*), hydroxysteroid 17 beta dehydrogenase 12 (*HSD17B12*), acetyl‐CoA acetyltransferase, mitochondrial (*ACAT1*), acyl‐CoA dehydrogenase medium chain (*ACADM*), acyl‐CoA dehydrogenase short/branched chain (*ACADSB*) and steroyl‐CoA desaturase (*SCD*). We performed protein–protein interaction (PPI) via network analysis using STRING database to confirm the interaction between these genes. As presumed, there was a strong PPI as shown by network analysis (Figure 2E).

Further, enrichment analysis from gene set enrichment analysis (GSEA) identified RNA transcription and oxidative stress to be positively correlated with knockdown NAT10, which confirmed the role of NAT10 in transcription and whose depletion could result in oxidative stress (Figure S3B). Additionally, we performed qRT‐PCR to validate the expression levels of the FA metabolic, which confirmed the downregulation of the FA metabolic genes in NAT10 depleted MCF7 cells (Figure 2F). We also confirmed FA metabolic gene expression in HeLa and other breast cancer cells; MDA‐MB‐231, MDA‐MB‐468 and T47D (Figure S3C). The expression levels of the studied genes were also downregulated in breast cancer cell lines 48 h posttreatment with Remodelin (the only known inhibitor of NAT10) (Figure S3D). Therefore, we deduce that NAT10 regulates FA metabolism.

Growing evidence has shown the crucial role of FA metabolism in metastasis and treatment resistance.^13^, ^14^, ^15^, ^16^, ^17^, ^18^, ^19^ The reprogramming of FA metabolism in cancer cells, especially in obese patients causes increased severity and poor survival. Therefore, exploring and targeting the FA metabolic pathway is crucial in understanding and treating cancer.^20^, ^21^, ^22^, ^23^, ^24^, ^25^, ^26^ Since the transcriptome of the FA metabolic genes is regulated by NAT10. We then ask if the transcripts of the FA metabolic genes are regulated by NAT10‐mediated ac4C modification.

### 2.3 Fatty acid metabolic genes are regulated by NAT10 mediated ac4C modification and stability

**FIGURE 3:**
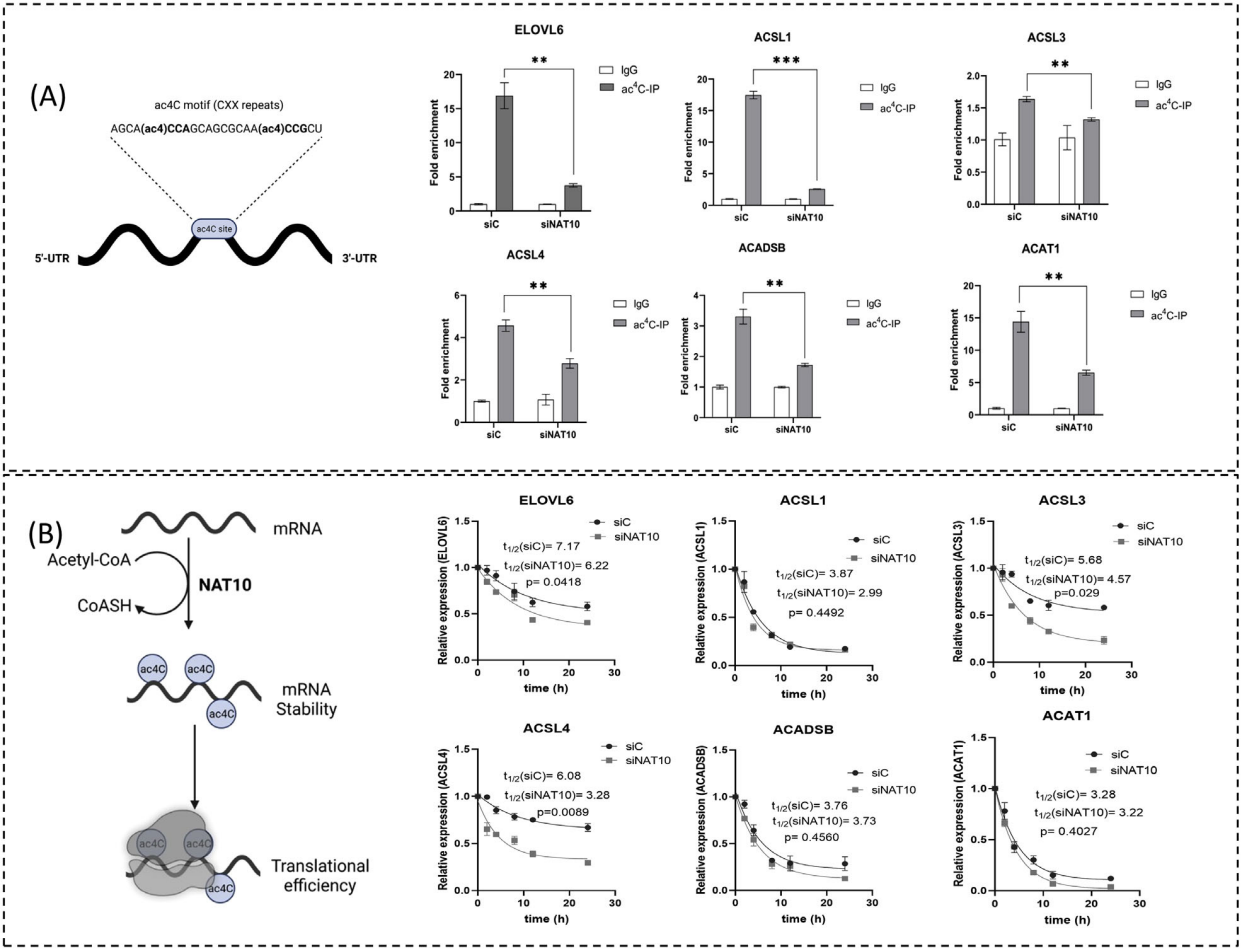
NAT10 regulates stability of fatty acid metabolic genes in ac4C dependent manner. (A) ac4C levels of RNA transcripts in MCF7 24 h after NAT10 knockdown. Error bars are represented as mean ± SEM (*n* = 3). **p < .01; and ***p < .001. (B) Stability assay of MCF7 24 h after NAT10 knockdown followed by treatment with actinomycin D. Analyses were performed using one‐phase decay in Graphpad prism version 8.0.1. *p*‐Value of ≤.05 are considered statistically significant.

To gain evidence for ac4C deposition on the FA metabolic genes’ RNA transcripts, we performed ac4C based RNA immunoprecipitation‐PCR (ac4CRIP‐PCR). First, we knockdown MCF7 cell line with NAT10 siRNA and pull down the RNA using the anti‐ac4C antibody using input and igG as controls. These samples were subject to qRT‐PCR. Exploring all the identified FA metabolic genes, we discovered that the ac4C levels on the RNA transcripts of *ELOVL6, ACSL1*, *ACSL3*, *ACSL4*, *ACADSB* and *ACAT1* were significantly depleted in NAT10 knockdown cells (Figure 3A). Hence, this result suggests that NAT10 regulates FA metabolic genes through ac4C‐dependent modification. To further understand the concept of NAT10‐mediated ac4C modification in FA metabolic genes, we performed mRNA stability assay using actinomycin D. Generally, the RNA transcripts of all studied genes showed decreased half‐life upon NAT10 knockdown, however, a significant difference was observed in the half‐life of *ELOVL6*, *ACSL3* and *ACSL4* (Figure 3B).

Based on the decrease in half‐life of the RNAs of the FA metabolic genes in NAT10 depleted cells, we presumed that NAT10 regulates the RNA stability of the studied FA metabolic genes. Notably, *EVOVL6*, *ACSL3* and *ACSL4* mRNA transcripts showed significant reduction in both ac4C levels and half‐life in NAT10 depleted MCF7. To assess the impact of reduced ac4C levels and half‐life of *EVOVL6*, *ACSL3* and *ACSL4* mRNA transcripts on their protein expression we therefore performed western blot. Interestingly, observable decrease in expression was observed in all three proteins: ELOVL6, ACSL3 and ACSL4 (Fig. S4).

Overall, our result showed that NAT10 regulates FA metabolism in ac4C‐dependent manner. Since fatty metabolism plays a crucial role in lipid accumulation, we assessed the impact of NAT10 on lipid accumulation.

### 2.4 NAT10 modulates palmitate‐driven lipid accumulation in cancer cells

**FIGURE 4:**
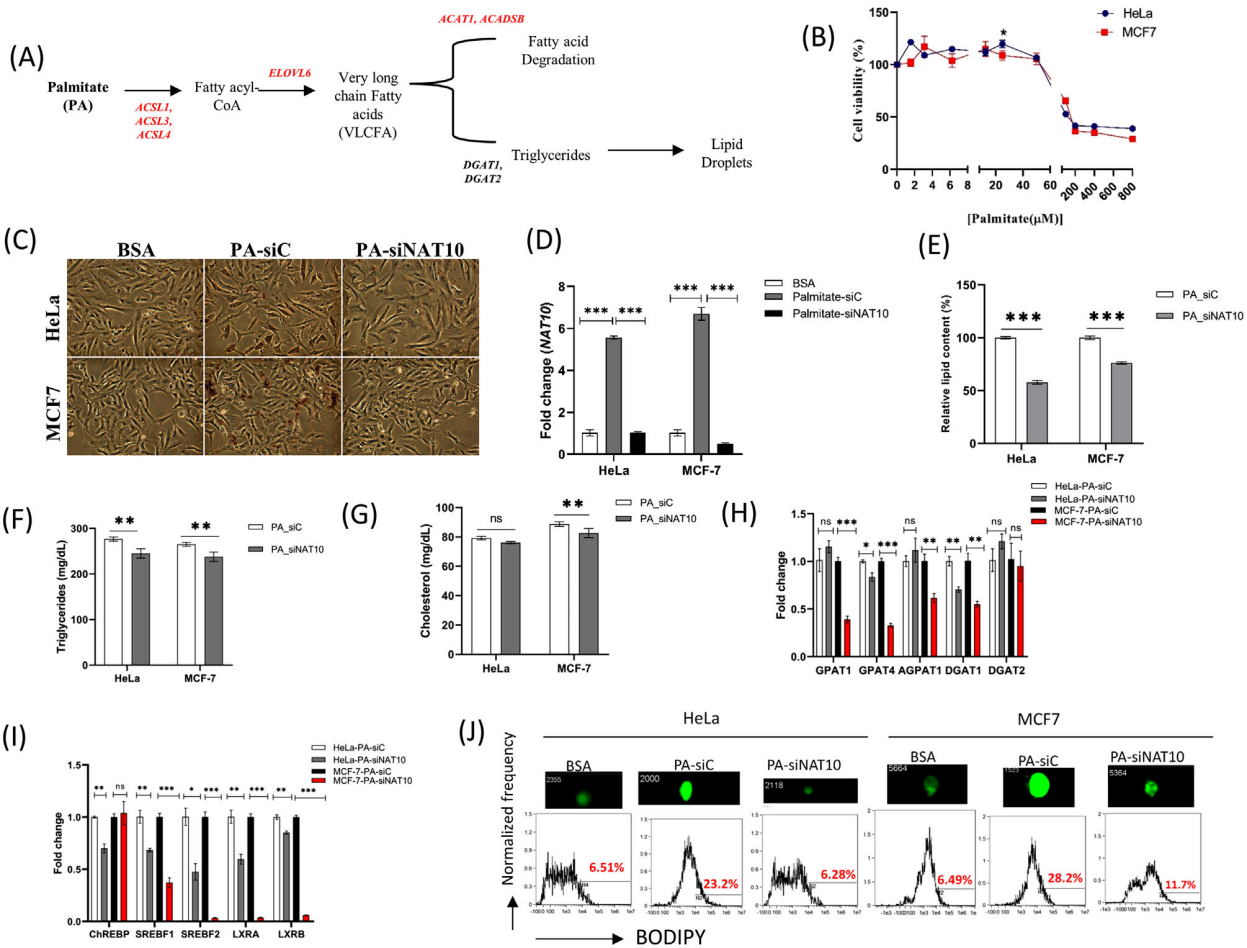
NAT10 regulates palmitate driven lipid accumulation. (A) Schematics of palmitate‐driven lipid accumulation. (B) Cell viability of cells loaded with palmitate after 24 h mean ± SEM (*n* = 3). (C) Oil red O staining of cells after 24 h loading with 25 μM of palmitate followed by NAT10 knockdown at 24 h, image magnification X40. (D) NAT10 expression levels after 24 h loading with 25 μM of palmitate followed transfection for another 24 h. (E–G) Lipid profile of cells loaded with 25 μM of palmitate followed by NAT10 knockdown at 24 h. (H,I) Gene expression for lipogenic related genes in palmitate loaded cells after knockdown using NAT10 siRNA. (J) Flow cytometery analysis of lipid droplets of cells loaded with palmitate followed by knockdown with NAT10 siRNA. All data are represented as mean ± SEM (*n* = 3). **p < .01; ***p < .001; and ns > .05.

Our hypothesis here is that since the studied FA metabolic genes could promote FA metabolism leading to lipid droplet formation, we test if the depletion of NAT10 could cause decrease in lipid accumulation (Figure 4A). To assess the impact of NAT10 on lipid accumulation, we performed exogenous palmitate uptake assay on both HeLa and MCF7 cells. Since we wanted to achieve the highest palmitate uptake without any causes of cell death in our study, we subjected both cells (HeLa and MCF7) to different doses of bovine serum albumin (BSA)–palmitate conjugate and measured their cell viability using MTT assay. Both cell lines from our MTT assay showed optimum palmitate uptake at 25 μM without any significant cause of cell death (Figure 4B). After optimising the conditions for palmitate loading in baseline palmitate loading (Figure S5A), we then subjected both HeLa and MCF7 to palmitate uptake and then NAT10 knockdown using siRNA. Assessing the cell survival of palmitate in NAT10‐depleted cells (PA‐siNAT10) compared to control (PA‐siC), we observed approximately 30% reduction of viable cells in PA‐siNAT10 (Figure S5B). The cell viability assay was further confirmed by expression in cell survival genes; *BCL2*, *p16* and *p21* (Figure S5C).

Images from oil red O staining showed noticeable decrease in PA‐siNAT10 compared to PA‐siC and vehicle (BSA) (Figure 4C). Observing decreased lipid accumulation in NAT10‐depleted cancer cells, we then measured NAT10 expression levels and lipid profile in palmitate‐loaded NAT10‐depleted cancer cells. Surprisingly, the expression levels of *NAT10* were significantly elevated in PA‐siC versus BSA, however, reduced NAT10 expression was observed in NAT10 expression in PA‐siNAT10 versus PA‐siC (Figure 4D), suggesting that NAT10 expression plays a crucial role in lipid accumulation. Lipid profile analysis from NAT10‐depleted cancer cells showed a significant decrease in overall lipid content, triglycerides (TAGs), and cholesterol levels of PA‐siNAT10 (Figure 4E–G). Based on the results obtained, we then decided to investigate the expression levels of selected genes essential for lipid accumulation and lipid droplet formation. Lipogenic genes, that is, *GPAT4* and *DGAT1* were significantly reduced in PA‐siNAT10, suggesting the role of NAT10 in reducing lipid accumulation (Figure 4H). However, while the expression of *AGPAT1* and *GPAT1* were significantly reduced in MCF7‐PA‐siNAT10; no reduction was recorded in HeLa‐PA‐siNAT10. To check if the results were based on cancer cells’ physiology; we assessed the expression of *AGPAT1* and *GPAT1* in other breast cancer cell lines; MDA‐MB‐231, MDA‐MB‐468 and T47D. Interestingly, all the three breast cancer cell lines showed a significant reduction in expression of *AGPAT1* and *GPAT1*, generally showing that the expression pattern in the two genes is peculiar to breast cancer cells and not HeLa cells (Figure S5D,E).

To further confirm if NAT10 is associated with lipid accumulation, we check the expression levels of transcription factors of lipogenic genes *ChREBP*, *SREBF1*, *SREBF2*, *LXRα*, and *LXRβ*. We also confirmed expression of lipogenic effector genes and transcription factors in baseline conditions; palmitate loaded (palmitate vs. BSA) and NAT10 knockdown (siNAT10 vs. siC) conditions (Figure S5F–I).

Results from the RT‐PCR of the lipogenic transcription factors showed significant downregulation in PA‐siNAT10 cancer cells, further providing strong evidence of NAT10 as a regulator of lipid accumulation (Figure 4I). Since all our experiments in palmitate‐loaded cancer cells showed significant decrease in biochemical and molecular mediators of lipid accumulation, we decided to measure the lipid droplet formation. Results from lipid droplet formation of both HeLa and MCF7 showed a drastic decrease in lipid droplets upon knockdown with NAT10 siRNA (Figure 4J). Overall, these findings suggest that NAT10 modulates lipid accumulation in cancer cells by regulating the functions of lipogenic genes.

### 2.5 NAT10 modulates fatty acid metabolism of palmitate‐driven cancer cells in ac4C‐dependent manner

**FIGURE 5:**
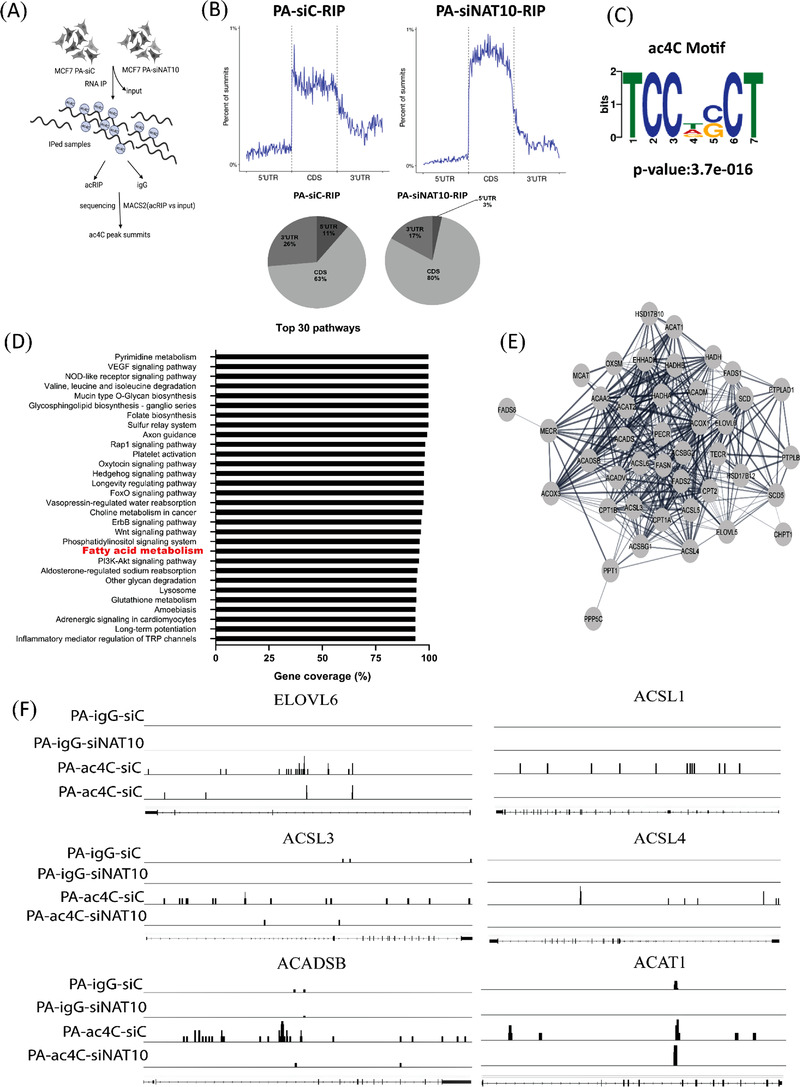
Transcriptome‐wide mapping of ac4C in NAT10‐depleted palmitate‐loaded cells. (A) Schematics of acRIP‐seq. (B) Percentages of summits based on location within coding sequence (CDS) or UTRs in acetylated transcripts of palmitate‐loaded control (PA‐siC‐RIP) vs. palmitate loaded with NAT10 knockdown (PA‐siNAT10‐RIP). (C) Enriched motif of ac4C peak from PA‐siNAT10. (D) Pathways identified from differential peaks in PA‐siC vs. PA‐siNAT10. (E) Network analysis showing the protein–protein interaction (PPI) fatty acid metabolic genes related with PA‐siNAT10. (F) Browser view of ac4C peaks in fatty acid metabolic genes; ELOVL6, ACSL1, ACSL3, ACSL4, ACADSB and ACAT1 mapped to the human reference genome.

**FIGURE 6:**
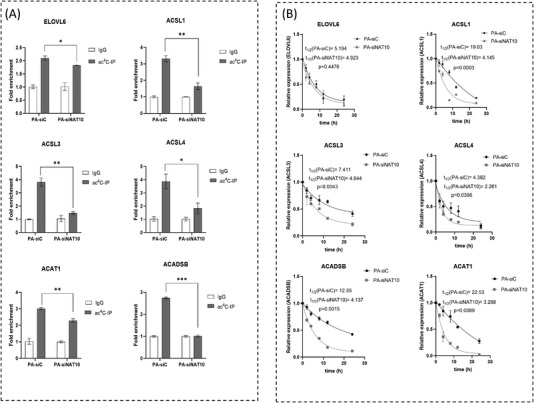
NAT10‐dependent ac4C validation acRIP‐seq in NAT10‐depleted palmitate loaded cells. (A) ac4C levels of RNA transcripts in NAT10‐depleted palmitate‐loaded cells. Error bars are represented as mean ± SEM (*n* = 3). **p < .01; and ***p < .001. (B) Stability assay of MCF7 24 h after NAT10 knockdown followed by treatment with actinomycin D. Analyses were performed using one‐phase decay in Graphpad prism version 8.0.1. *p*‐Value of ≤.05 are considered statistically significant.

To understand the impact of ac4C on palmitate‐driven lipid accumulation, we performed acetylated RNA immunoprecipitation sequencing (acRIP‐seq) (Figure 5A). Like previous studies from acRIP‐seq, our result showed overall ac4C reduction at the 5'UTR in palmitate‐loaded cells transfected with NAT10 siRNA.^27^ However, we noticed an increase of ac4C in the coding region site (CDS) region; this could be due to the influence of palmitate loading in cells (Figure 5B). Sequential analysis of ac4C peaks showed that TCCDSCT was highly enriched within the ac4C sites, which is same as the CXX repeats as suggested from previous studies (Figure 5C).^1^, ^28^ Based on ac4C peak annotation, we identified enriched pathways based on percentage coverage (Table S2). Approximately 96% of the FA metabolic gene transcripts were enriched, suggesting that NAT10 regulates FA metabolism in palmitate‐loaded cancer cells in an ac4C‐dependent manner (Figure 5D). Further, network analysis showed that all the NAT10‐mediated ac4C FA metabolic gene transcripts undergo PPI interaction (Figure 5E). Next, we asked if previous FA metabolic genes from our RNA seq; ELOVL6, ACSL1, ACSL3, ACSL4, ACADSB and ACAT1 were also enriched in palmitate‐loaded conditions in ac4C‐dependent manner. Surprisingly, a noticeable decrease in ac4C peaks were seen in PA‐siNAT10 cancer cells via browser shots, suggesting that the genes were also modulated by NAT10‐dependent ac4C modification (Figure 5F). To validate our acRIP‐seq, we performed RIP‐PCR and mRNA stability assay to check the impact of NAT10 on the stability of genes in NAT10‐depleted cells loaded with palmitate (Figure 6). Interestingly, all the FA metabolic genes showed significant ac4C reduction in PA‐siNAT10, confirming results from Figure 5F as reported in acRIP‐seq (Figure 6A). Additionally, in the PA‐siNAT10 condition, the mRNA stability of all half‐lives was significantly reduced; this could be due to the effect of NAT10 on the mRNA stability of FA metabolic genes. We noticed a higher impact of NAT10 on stability in PA‐siNAT10 (with palmitate) compared to siNAT10 (without palmitate); this distinctive difference in the mRNA stability could be due to loading of PA‐siNAT10 cells with exogenous palmitate, which might have played a crucial role in promoting palmitate‐driven FA metabolism.

Cumulative evidence from RNA‐seq and acRIP‐seq showed FA metabolic genes were regulated via NAT10‐dependent ac4C modification; therefore, we decided to perform untargeted lipidomics based on this evidence.

### 2.6 NAT10 alters global lipidomic landscape in cancer cells

**FIGURE 7:**
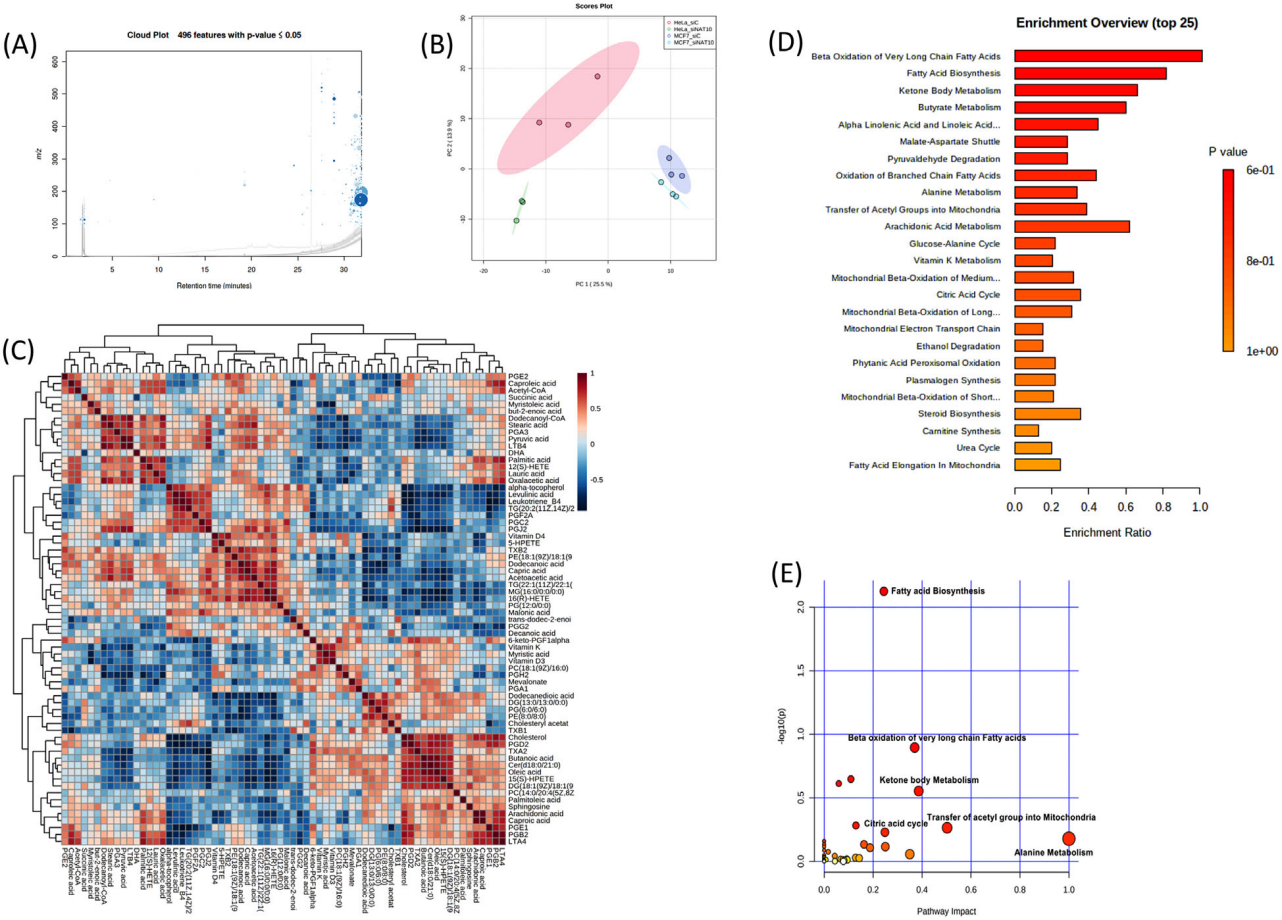
Impact of NAT10 on lipid metabolism in cancer cells. (A) Cloud plot of lipid features identified in NAT10 knockdown HeLa and MCF7. (B) Principal correlation analysis (PCA) analysis of HeLa and MCF7 transfected with NAT10 siRNA (siNAT10) vs. control (siC). (C) Correlation analysis of lipid species of siNAT10 vs. siC in HeLa and MCF7. (D) Enrichment analysis from lipidomes in NAT10 knockdown HeLa and MCF7. (E) Pathway analysis generated from lipidomes in NAT10 knockdown HeLa and MCF7.

All our previous findings suggest that NAT10 plays a crucial role in FA metabolism and lipid accumulation. To acquire evidence of this concept, we performed high‐throughput untargeted lipidomics in NAT10 knockdown cancer cells. In untargeted lipidomics, 496 out of 2 279 lipids were statistically significant in NAT10 knockdown (Figure 7A, Table S3). Lipidomes were acquired using data‐dependent acquisition (DDA) mode followed by processing with XCMS database. To identify the lipids, we used LIPID MAPS^®^ (https://www.lipidmaps.org/). Using Metaboanalyst 5.0 (https://www.metaboanalyst.ca/), we performed the principal correlation analysis (PCA), correlation and enrichment analysis of lipidomes (Figure 7B–E). PCA showed significant variation in siNAT10 VS siC HeLa and MCF7 cells suggesting variation in the profiles of lipid species between the two groups of both cell lines (Figure 7B). Majority of the identified lipid species showed significant correlation demonstrating the similarity in the features of the identified lipids (Figure 7C). Small molecule pathway database (SMPD) was used to map and identify enrichments and pathways analyses of lipid species. Top pathways based on enrichment analysis of lipid species were beta‐oxidation of very long‐chain fatty acids (VLCFA), FA biosynthesis, ketone body metabolism, butyrate metabolism and alpha‐linolenic acid and linolenic acid metabolism (Figure 7D). Notably, many other enriched pathways were mitochondrial beta‐oxidation of long‐ and short‐chain FAs and FA elongation from mitochondria which were similarly identified in our previous study on metabolomic impact of Remodelin, a small molecular inhibitor of NAT10 on cancer cells.^12^ Pathway analysis showed a similar pattern with enrichment analysis based on significance. The most significant pathways from the identified lipid species were FA biosynthesis, beta‐oxidation of VLCFA and ketone body metabolism (Figure 7E). Taken together, the identified pathways from enrichment and pathways analysis were related to FA metabolism, thereby supporting our previous studies confirming the role of NAT10 in FA metabolism and lipid accumulation. The list of top enriched lipid sets, and their members identified in our studies are depicted in Table 1.

**TABLE 1:** Enriched pathways obtained from untargeted lipidomics of NAT10 knockdown cancer cells

			**Peak intensity status**	
**Enriched lipid sets**	**Total/hits**	**Lipids**	**HeLa**	**MCF7**
Beta oxidation of very long chain fatty acids	17/6	Capric acid	Down	Down
		Caproic acid	Down	Down
		Acetylcarnitine	Down	Down
		Acetyl‐CoA	Down	Down
		Dodecanoic acid	Down	Down
		Dodecanoyl‐CoA	Down	Down
Fatty acid Biosynthesis	35/10	Butanoic acid	Down	Down
		Acetoacetic acid	Down	Down
		Capric acid	Down	Down
		Caproic acid	Down	Down
		Dodecanoic acid	Down	Down
		Malonic acid	Down	Down
		Myristic acid	Down	Down
		Acetyl‐CoA	Down	Down
		trans‐dodec‐3‐enoic acid	Down	Down
		Palmitic acid (Palmitate)	Down	Down
Ketone body metabolism	13/3	Acetoacetic acid	Down	Down
		Succinic acid	Down	Down
		Acetyl‐CoA	Down	Down
Butyrate metabolism	19/4	Butyric acid	Down	Down
		Acetoacetic acid	Down	Down
		Succinic acid	Down	Down
		Acetyl‐CoA	Down	Down
Alpha linolenic acid and linolenic acid metabolism	19/3	α‐linolenic acid	Down	Down
		Linolenic acid	Up	Down
		Arachidonic acid	Down	Down

Total/hit is the ratio of total number of lipid species available in each enriched pathway over the lipid species identified from our study. The down or up peak intensity status is based on peak intensities of NAT10 knockdown (siNAT10) vs. transfection ctrl (siC).

### 2.7 Lipidomics confirms NAT10 as regulator of fatty acid metabolism

**FIGURE 8:**
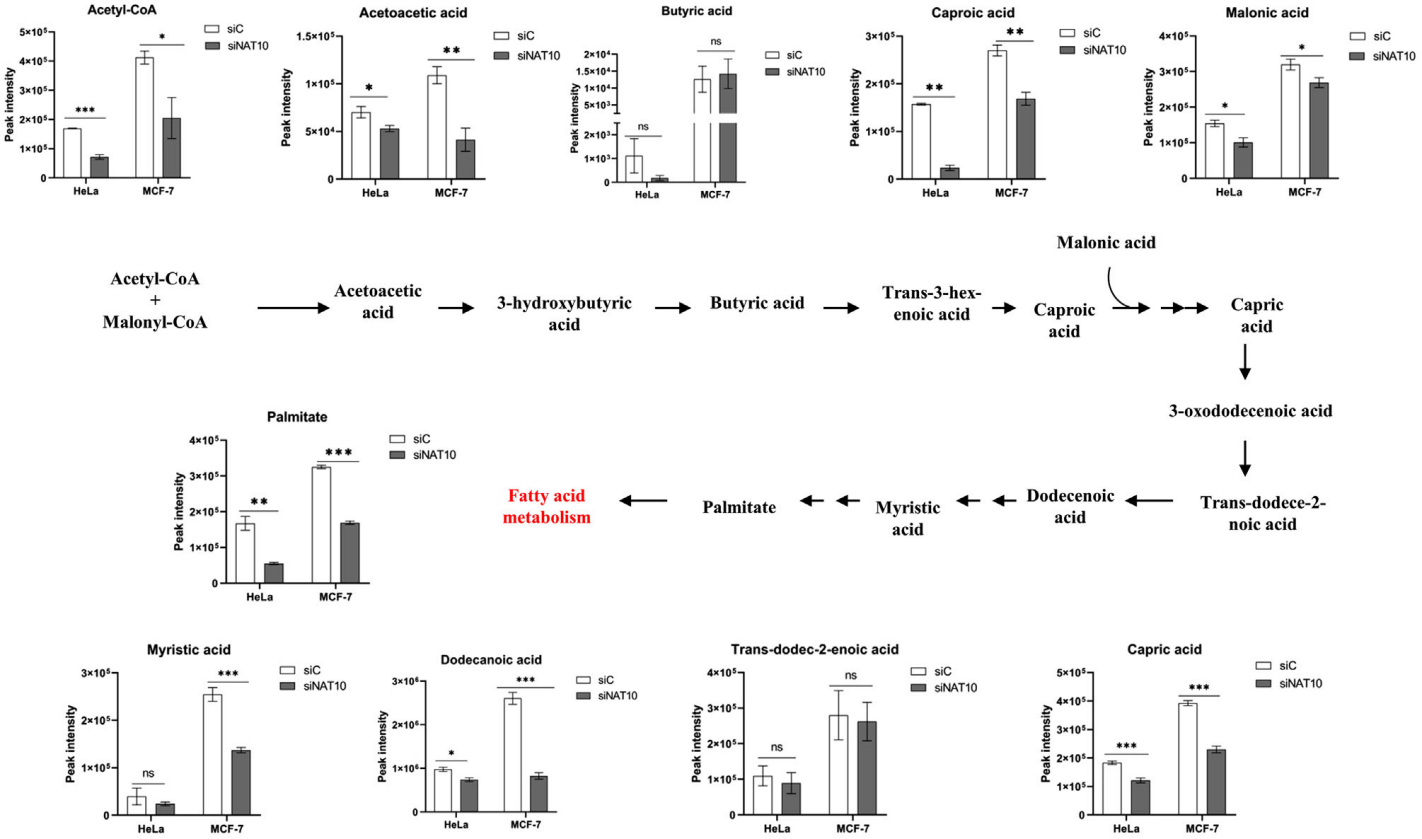
NAT10 knockdown reduces fatty acid metabolism in cancer cells. The metabolic intermediates showed significant decrease in peak intensities post transfected with NAT10 siRNA. Graph of metabolic intermediates are represented as mean ± SEM (*n* = 3). **p < .01; ***p < .001; and ns > .05.

Through its metabolic intermediates, *de novo* FA metabolism plays a crucial role in metabolic reprogramming of cancer cells, cell proliferation, cell survival and cancer progression.^25^, ^29^, ^30^, ^31^ Our lipidomics data showed a significant decrease in peak intensities of metabolic intermediates such as acetyl‐CoA, acetoacetic acid, butyric acid, caproic acid, malonic acid, capric acid, dodecanoic acid, trans‐dodec‐2‐enoic acid, myristic acid and palmitate (Figure 8). Decreased peak intensity of palmitate from our lipidomics further provided evidence that NAT10 modulates palmitate‐driven lipid accumulation. Among the metabolic intermediates that we decreased upon NAT10 depletion is acetyl‐CoA. Notably, acetyl‐CoA is the substrate for NAT10‐mediated RNA acetylation. Therefore, the reduced peak intensity of acetyl‐CoA is consistent with decreased global ac4C level in NAT10 depleted cancer cells, which was measured via dotblot. Acetyl‐CoA is significant for FA metabolism and is a central molecule for other metabolic pathways such as tri‐carboxylic acid (TCA) cycle, lipid formation, energy expenditure, protein metabolism, carbohydrate metabolism, etc. Overall, our study further provided strong evidence of the impact of NAT10 on FA metabolism and lipid accumulation.

**FIGURE 9:**
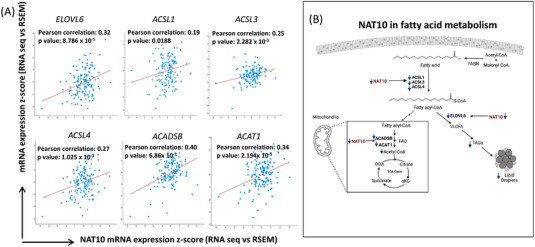
(A) Correlation analysis of fatty acid metabolic genes against NAT10 in breast cancer retrieved from cBioportal webserver. (B) Proposed mechanism of NAT10 as a regulator of fatty acid metabolism through mitochondrial lipid metabolism and lipid droplets formation.

Since all evidence showed a strong relationship between NAT10 and FA metabolism, we then explore the correlation of NAT10 to the studied FA metabolic genes in breast cancer using web server for cBioportal (https://www.cbioportal.org/). Notably, all the FA metabolic genes *ELOVL6*, *ACSL1*, *ACSL3*, *ACSL4*, *ACDSB* and *ACAT1* showed significant positive correlation with NAT10 suggesting that overexpression of NAT10 in breast cancer patients is associated with FA metabolism (Figure 9A). Elevated FA metabolism in breast cancer has since been associated with poor survival, metastasis and increased cancer cellular & tissue growth.

Overall, our proposed mechanism is a deficiency of NAT10 leads to decreased levels of *ACSL1*, *ACSL3*, *ACSL4*, *ELOVL6*, *ACDSB* and *ACAT1* (Figure 9B).

Decreased *ACSL1*, *ACSL3 and ACSL4* implicate in reduced levels of fatty acyl‐CoA formation, which is crucial for lipid droplet formation and fatty acid oxidation (FAO). Reduced *ELOVL6* negatively regulates the conversion of fatty acyl‐CoA to VLCFA which leads to formation of triglycerides and lipid droplets. Additionally, reduced expression levels of *ACADSB* and *ACAT1* prevent acetyl‐CoA formation through FAO. Overall, NAT10 depletion in cancer cells could cause complete blockage of FA metabolism, resulting in reduced cell growth and cell death.

## 3 DISCUSSION

NAT10 plays a crucial role in carcinogenesis through influencing EMT, hypoxia, ribosomal biogenesis and overall, promoting translational efficiency. Recently, we reported that treating cancer cells with Remodelin, a small molecule inhibitor of NAT10, causes alteration in global lipid metabolism. Whether NAT10 facilitates other important pathways in cancer cells is unknown. Our study showed that NAT10 promotes FA metabolism in cancer cells by stabilising the mRNA transcripts of FA metabolic genes via ac4C mediated modification. This concept was also confirmed using palmitate uptake assay.

Cancer cells require biomolecules such as nucleic acid, protein, and lipids for growth and proliferation.^32^, ^33^ In both normal and cancer cells, lipid species such as TAGs, Diacylglycerols (DAGs), cholesterol, phospholipids and FAs are crucial for energy supply and membrane structural integrity ^34^, ^35^. Additionally, lipids serve as signalling molecules in biological processes, including cell survival, cell growth and differentiation. Here, we found that NAT10 depletion implicates reducing the lipid levels, which could be why morphological changes, reduced cell proliferation and growth were observed in NAT10 depleted cancer cells.

FA synthesis, uptake, degradation and storage are cellular activities essential for cancer cell survival. FASN catalyzes the synthesis of FAs, and elevation in the expression of FASN is associated with cancer progression in breast and ovarian cancer.^36^ For FA to be processed for storage or degradation, it is activated by ACSL1, ACSL3, and ACSL4, whose action determines the fate of intracellular free FAs.^25^, ^37^, ^38^, ^39^ Overexpression of the ACSL family in cancer is associated with TNFα‐mediated pro‐inflammatory activities, cancer progression, and poor prognosis.^40^ Furthermore, overexpression of the ACSLs is reported in many cancer types such as colon, liver and breast cancer.^25^, ^37^, ^39^, ^41^, ^42^ RNA‐seq data from our study showed decreased expression of ACSL1, ACSL3 and ACSL4 in NAT10‐depleted cancer cells, which further confirmed that the mRNA transcripts of these genes are controlled by NAT10‐dependent ac4C mRNA acetylation.

The present study also showed that ELOVL6 is regulated via NAT10‐dependent ac4C mRNA acetylation. FAs committed to metabolism require ELOVL6 to elongate FAs leading to the formation of very long chain fatty acids (VLCFAs). The elongation process is critical in forming different subsets of FAs with diverse functions, from building blocks of cells to energy currencies or storage units. Committed to storage, the FAs react with glycerols in the presence of AGPAT, GPAT and DGAT to produce MAGs, DAGs and TAGs whose aggregation leads to the formation of lipid droplets or follow an alternative pathway to synthesise cholesterol in the presence of Hydroxymethylglutaryl‐CoA synthase. Our findings have shown that expression of *GPAT4* and *DGAT1* were reduced in NAT10‐depleted cancer cells and palmitate‐loaded NAT10‐depleted cancer cells suggesting that NAT10 depletion implicates in reduced lipid droplet formation. Evidently, the result was also observed in lipid droplet assay with BODIPY. Lipid accumulation in cancer cells influences tumour invasion and metastasis, leading to poor survival and resistance to conventional anti‐cancer treatment.^32^

During prolonged cellular starvation, FAs undergo degradation to meet cellular energy demand and utilisation; this process is achieved via the oxidation of FA to acetyl‐CoA in the presence of ACAT1 and ACADSB. Like FA synthesis, FA degradation is explored by cancer cells to compensate for loss energy during cell growth, cell proliferation and metastasis.^43^, ^44^, ^45^, ^46^

Acetyl‐CoA is a central metabolic intermediate for the TCA cycle, leading to the formation of NADH and FADH important for electron transport chain and oxidative phosphorylation, ultimately forming ATPs. Similarly, acetyl‐CoA is the substrate for NAT10‐dependent ac4C RNA acetylation. Our findings have shown that NAT10 depletion is associated with decreased global ac4C levels as well as acetyl‐CoA. Therefore, we presume that the presence of NAT10 drives the availability of acetyl‐CoA. Since ACAT1 and ACADSB are regulated via NAT10‐dependent ac4C RNA acetylation, thus, availability of NAT10 could lead to increased production of acetyl‐CoA through FA degradation.

Thus, our study provides for the first time the crucial role of NAT10‐dependent ac4C modification in regulating FA metabolism and lipid accumulation in cancer cells. Our findings also confirmed that FA metabolic genes and their metabolic intermediates are crucial for cancer survival and cell proliferation. NAT10 depletion in cancer cells results in the dysfunction of FA metabolism, causing cell death. Therefore, our study suggests that targeting NAT10 is a promising strategy for treating cancer.

The worldwide new cancer cases are estimated to be 19.3 million. Breast cancer is now the most commonly diagnosed cancer, with approximately 2.3 million new cases and a death rate of 600 000^47^. Hence in‐depth study is required to identify more efficient and effective treatment strategies. In conclusion, we showed that NAT10 depletion reduces the expression and stability of FA metabolic genes whose functions were known to significantly impact cancer progression, metastasis, and resistance to conventional anti‐cancer treatment. Therefore, targeting NAT10‐mediated ac4C modification in cancer shows promising therapeutic benefits.

## 4 MATERIALS AND METHOD

### 4.1 Cell culture

Human cervix cell carcinoma cells, HeLa and breast cancer cells, MCF‐7, MDA‐MB‐231, MDA‐MB‐468 and T47D were cultured in Dulbecco's Modified Eagle Media (DMEM) (BIS BioTech, KSA) containing 10% fetal bovine serum (FBS) (Gibco, USA), 1% penicillin–streptomycin (MOLEQULE‐ON®, New Zealand). All studied cells were maintained in humidified incubator conditions of 37°C and 5% CO_2_. In this study, cells were grown to 60%–70% confluence before transfection.

### 4.2 NAT10 siRNA transfection

Transfection was performed using siRNA targeting NAT10 (Santa Cruz Biotechnology Inc, USA) by Lipofectamine™ RNAiMAX (Invitrogen, USA) following the manufacturer's instruction. Briefly, lyophilised NAT10 siRNA was reconstituted using RNAse free water to obtain a stock solution of 10 μM. Cells were seeded in six‐well plate for 24 to attain 50%–60% confluence and then treated with 60 nM of NAT10 siRNA or scramble control.

### 4.3 RNA ac4C dot blot

RNA dot blot was performed as previously described. Briefly, total RNA were placed at 65°C for 5 min and immediately cooled on ice for 3 min. Denatured RNAs were then spotted on the Hybond N+ membrane and crosslinked with UV light. The ac4C modifications on RNA were detected using anti‐ac4C (Abcam).

### 4.4 RNA sequencing

#### 4.4.1 RNA preparation extraction

Total RNA was isolated using the TRI reagent (Sigma, USA) according to the manufacturer's protocol. Then, we evaluated the RNA purity using a NanoPhotometer^®^ spectrophotometer (DeNovix, USA).

#### 4.4.2 Library construction, quality control and sequencing

From the total RNA, the messenger RNA was isolated and purified using poly‐T oligo‐attached magnetic beads. After fragmentation, the cDNA strand was synthesised using random hexamer primers, followed by cDNA dA‐tailing. Tailed cDNAs were ligated to sequencing adaptors and then library construction.

The library was checked with Qubit 2.0 and real‐time polymerase chain reaction (PCR) for quantification and bioanalyser for size distribution detection. Quantified libraries were pooled and sequenced on Illumina HiSeq/NovaSeq platform, according to effective library concentration and data amount. Raw data (raw reads) of fastq format were firstly processed through in‐house perl scripts. In this step, clean data were obtained by removing reads containing adapter, reads containing poly‐*N* and low‐quality reads from raw data. All the downstream analyses were based on the clean data with high quality. Index of the reference genome was built and paired‐end clean reads were aligned to the reference genome using Hisat2 v2.0.5. The featureCounts v1.5.0‐p3 was used to count the reads numbers mapped to each gene and then fragments per Kilobase of transcript per million mapped fragments of each gene was calculated based on the length of the gene and reads count mapped to gene.

#### 4.4.3 Differential gene expression and enrichment analysis

Differential expression analysis was performed using the DESeq2Rpackage (1.20.0). DESeq2 provide statistical routines for determining differential expression in digital gene expression data using a model based on the negative binomial distribution. The resulting *p*‐values were adjusted using the Benjamini and Hochberg's approach for controlling the false discovery rate (FDR). Genes with *p*‐value ≤.05 found by DESeq2 were assigned as differentially expressed.

Gene ontology (GO) enrichment analysis of DEGs was implemented by the clusterProfiler R package, in which gene length bias was corrected. GO terms with corrected *p*‐value less than.05 were considered significantly enriched by differential expressed genes. KEGG database was used to identify enriched pathways by DEGs (http://www.genome.jp/kegg/).

### 4.5 Real‐time quantitative PCR

Transfected and palmitate loaded HeLa and MCF7 cells were harvested and washed two times with ice‐cold phosphate‐buffered saline (PBS). Total RNA was extracted by TRI Reagent® solution (Ambion) following the manufacturer's instructions. The purity and concentration of the extracted RNA were determined at 260/280 nm using a spectrophotometer (nanophotometer) and were reverse transcribed into cDNA with a High‐Capacity cDNA conversion Kit (Applied Biosystems) using 1 μg of total RNA. Gene expression was determined by qRT‐PCR performed with SYBR Green (Applied Biosystems). The PCR program started at the preheating step of 50°C for 2 min, 95°C for 2 min followed by 40 cycles of denaturation at 95°C for 15 s, annealing at 60°C for 1 min, and a final extension at 60°C for 1 min. Melting curves were obtained at 60°C. Data were reported as fold change (2^−△△^ *^Ct^*). Assays were performed independently in triplicate. Details of primer sequences used RT‐PCR can be found in Table S4.

### 4.6 ac4C RNA immunoprecipitation

NAT10 knockdown cells were washed with iced cold PBS; trypsinised and pelleted by centrifugation at 1 500 rpm for 5 min at 4°C. Cell pellets were lysed with RIP buffer (300 mM Tris‐HCl, 150 mM KCl, 0.5 mM fresh DTT, 0.5% (v/v) NP40, ×1 protease inhibitor) and incubated in −80°C for 24 h. Lysate was then centrifuge at 14 000 rpm for 10 min at 4°C, then 10% of input sample was collected prior to adding 2 μg of either Anti‐ac4C (Abcam) or corresponding anti‐IgG (Cell Signaling). Contents were allowed to incubate overnight at 4°C with constant gentle rotation. Next, protein A/G agarose beads (Cell Signaling) was added and allowed to rotate for another 2 h. Beads were collected after centrifugation at 1 000 rpm for 2 min. The RNA of input and IPed samples were extracted with Tri Reagent^®^ solution (Ambion) and subjected to RT‐PCR. Primers for RIP‐PCR were designed based of the CXX motif with the aid of prediction of N4‐acetylcytidine (ac4C) modification sites in mRNA (PACES) (http://www.rnanut.net/paces/) ^48^ Details of primer sequences used for RIP‐PCR can be found in Table S5.

### 4.7 Half‐life and mRNA stability assay

Cells were seeded in six‐well plate for 24 h followed by treatment with 5 μg/ml actinomycin D. Cells are then collected at different time points. Total RNA was extracted with Tri Reagent® solution (Ambion) and the mRNA levels of studied genes were determined using RT‐PCR. The half‐life and turnover rate were calculated according to previous reports.^11^

### 4.8 Preparation of BSA–palmitate conjugate

Approximately 25.6 mg of palmitate was dissolved in 1 ml of 0.1N NaOH at 70°C until the solution is clear. Simultaneously, 500 mg of FA free BSA (FFA–BSA) was dissolved in 5 ml distilled water and stored at 4°C until it is completely dissolved. 3.7 ml of BSA solution is then equilibrated at 55°C followed by the addition of 301 μl palmitate solution with stirring between each hour until a clear conjugate solution is achieved. The prepared conjugate is then filtered and stored in −20°C until ready for use.

### 4.9 Palmitate loading and cell viability

Cell viability was assessed by MTT (3‐[4,5‐dimethylthiazol‐2‐yl]‐2,5 diphenyl tetrazolium bromide). Briefly, HeLa and MCF‐7 cells (10 000 cells/well) were seeded in 96‐well plate for 24 h to allow them to adhere to the plate, then loaded with 1.56–800 μM bovine serum albumin–palmitate (BSA–PA). After 24 h post‐loading, 10 μl solution containing MTT (5μg/ml, Invitrogen) was added to the wells and incubated for 3 h. After the incubated time, DMEM medium containing MTT was removed from the wells, 100 μl Dimethylsulfoxide was subsequently added to each well and allowed to incubate for atleast 30 min at 37°C. The absorbance of content was read with a microplate reader (BioTek^®^, USA) using the Gen5^™^ software for microplate reading and data analysis.

Cell viability was graphically represented in mean±SEM values were calculated using GraphPad Prism version 8.0.1.

### 4.10 Flow cytometry

Cells were treated in various conditions; either NAT10 knockdown, palmitate loading or both. Treated cells were trypsinised, washed, fixed with 4% formaldehyde and subjected to different flow cytometric analysis.

Apoptosis assay was performed with Annexin V‐FITC and propidium iodide (PI, 5 μg/ml) acquired at 2 000 cells per sample. Cell cycle assay was conducted with 5 μL PI (5μg/ml) acquired at 5 000 cells per sample. Mitochondrial membrane potential and dead cell assay was performed with JC‐1 (2 μM) and 5 μL PI (5 μg/ml) acquired at 5 000 cells per sample. Stained cells were analysed using Guava® easyCyte Flow cytometer.

Lipid droplets formation was measured via staining with BODIPY 493/503. The assay was acquired at 10 000 cells per sample using Amnis Flowsight.

### 4.11 Lipid content analysis

The cellular lipid content of transfected and palmitate load cells was measured via Oil Red O staining (Sigma, USA).^12^ After 24 h, transfected and palmitate‐loaded cells were washed two times with PBS, fixed in 10% formalin for 30 min, followed by staining with Oil Red O and then incubated for an additional 30 min at room temperature. Oil Red O stain was removed, and wells were washed with iced cold distilled water and images from the treated cells were recorded with a light microscope (Nikon, USA). Next, 300 μl isopropanol was added to each well. Treated cells were allowed to incubate at room temperature for 5 min with gentle shaking to remove stained lipids with Oil Red O. Then, 100 μl of the eluate from each well was transferred to a 96 well plate, and the absorbance value was recorded at 490 nm wavelength using microplate reader (BioTek®, USA) with Gen5™ software.

Lipid content was extracted from transfected and palmitate load cells according to the Folch method. Briefly, cells were added to 1 ml of chloroform: methanol (2:1) and vortexed, followed by the addition of 200 μl distilled water. The mixture was then vortexed vigorously and centrifuged at 12 000 rpm for 10 min to generate an upper aqueous phase and lower organic phase. From the lower organic phase, 300 μl was placed into an already weighed 1.5 ml Eppendorf tube and dried overnight at room temperature. The dried lipid content was dissolved in 100% isopropanol. The triglycerides and total cholesterol were measured using biochemical assay kits (Crescent diagnostics, Jeddah, Saudi Arabia).^49^

### 4.12 ac4C RNA immunoprecipitation sequencing (acRIP‐seq)

Cells were washed with ice‐cold PBS; trypsinised and pelleted by centrifugation at 1 500 rpm for 5 min at 4°C. Cell pellets were then lysed with RIP buffer and incubated in −80°C for 24 h. Lysates were centrifuged at 14 000 rpm for 10 min at 4°C, followed by collection of 10% input sample prior to adding 2 μg of either anti‐ac4C (Abcam) or corresponding anti‐IgG (Cell Signaling). Content was allowed to incubate overnight at 4°C with constant gentle rotation. Next, protein A/G agarose beads (Cell Signaling) was added and allowed to rotate for another 2 h. Beads were collected after centrifugation at 1 000 rpm for 2 min. The RNA of input and IPed samples were extracted with Tri Reagent® solution (Ambion) and subjected to sequencing.

#### 4.12.1 Sample test, library preparation and sequencing

IPed RNA samples were detected using Agilent 2100 to determine fragmentation distribution and purity was checked Agarose gel electrophoresis.

High‐quality samples were then fragmented into 250 bp and then reverse transcribed to cDNA with random primer. The cDNA was then dA‐tailed and ligated to sequencing adaptors and then library formation via PCR amplification.

Library constructs were initially quantified using Qubit 2.0. Accurate library concentration was quantified using q‐PCR (library effective concentration >2 nM) to ensure standard library quality. Samples that passed the library quality control check were then subjected to sequencing using Illumina HiSeq/NovaSeq platform.

The original data from the sequencing platform were then transformed to sequencing reads called Raw data. Raw data were recorded of fastq file which is then trimmed using Skewer 0.1.126 and then mapping of different genome characters with BWA 0.7.12.

#### 4.12.2 Peak annotation and enrichment analysis

The peak calling is done using MACS2 (threshold *p*‐value ≤ .05). The number of peaks, the peak width and its distribution were calculated, and the peak‐related genes were discovered. MEME and Dreme software were used to detect significant motif sequence in the peak. Tomtom software was utilised to annotate motifs by mapping them to the annotated Motif database. Sequence logos were used to show the base bias in different positions in the binding sites in long Motif (8–30).

The peak distribution was conducted to predict ac4C binding sites. The immunoprecipitation effect was estimated according to ac4C binding sites. The ac4C‐mediated mechanism or function was predicted according to its binding character. Differentially analysis of peaks was performed using diffbind followed by GO enrichment analysis using GOseq/top GO Bioconductor 2.13 and for pathway enrichment analysis KOBAS 3.0 was used.

### 4.13 Lipid extraction

Transfected HeLa and MCF7 cells were harvested and washed two twice with ice‐cold PBS. Lipid was extracted using 1 000 μl of extracting solution made using ice‐cold methanol, acetonitrile, choloroform and water at ratio of 2:2:2:1 (v/v). The lower phase containing lipid was then isolated and air dried using rotary evaporator. Dried lipids were resuspended in N,O‐Bis(trimethylsilyl)trifluoroacetamide (BSTFA) containing 1% trimethylsilyl choloride (TMCS). Lipids were mixed well and heated to 60°C for up to 30 min until all liquid content is evaporated. Followed by cooling at room temperature and then resuspended in chloroform. Derivatised lipids were injected into gas chromatography‐tandem mass spectroscopy (GC‐MS; 7000C GC/MS Triple Quad, Agilent Technologies).

### 4.14 GC–MS analysis

Lipid profiles from NAT10 knockdown cells were processed using XCMS database (https://xcmsonline.scripps.edu/landing_page.php?pgcontent = mainPage). The processed lipidomes were then identified using LIPID MAPS^®^ (https://www.lipidmaps.org/). The PCA, enrichment and pathway analysis were performed using Metaboanalyst 5.0 (https://www.metaboanalyst.ca/).

### 4.15 Western blot analysis

Western blot was performed according to the standard method. Total protein was extracted using RIPA buffer (Cell Signaling Technology, USA), quantified using BCA protein assay kit (Sigma, USA) and normalised. Proteins were fractionated by Sodium dodecyl sulfate‐polyacrylamide gel electrophoresis (SDS‐PAGE) and transferred to nitrocellulose membrane. Nitrocellulose membrane was blocked using blocking solution (5% non‐fat milk in Tris buffered saline with Tween‐20 [TBST]) for 2 h, followed by overnight incubation with primary antibodies at 4°C. Next day, membrane was washed with TBST and incubated with secondary antibody containing horseradish peroxidase (HRP). Blots on membrane were detected using chemiluminescence (ECL). Antibodies used were ACSL3 (20710‐1‐AP, Proteintech), ACSL4 (22401‐1‐AP, Proteintech), ELOVL6 (21160‐1‐AP, Proteintech) and secondary antibody (7074S, Cell Signaling Technology, USA).

### 4.16 Statistical analysis

Data are recorded in mean ± SEM. All results of *p*‐value ≤.05 are considered statistically significant. Two‐tailed student *t*‐test was used for comparison between two groups. For multiple comparisons, Tukey multiple comparison was used. All statistical analysis was performed using Graphpad prism version 8.0.1 (GraphPad Software, La Jolla, CA).

### AUTHOR CONTRIBUTIONS

Conceptualisation, Mohammad Imran Khan; methodology, Mahmood Hassan Dalhat, Mohammed Razeeth Shait Mohammed, Hind Ali Alkhatabi, Aamir Ahmad, and Mohd Rehan; software, Mahmood Hassan Dalhat and Mohammed Razeeth Shait Mohammed; validation, Mahmood Hassan Dalhat, Mohammed Razeeth Shait Mohammed, Mohd Rehan, Hani Choudhry and Mohammad Imran Khan; formal analysis, Mahmood Hassan Dalhat, and Mohammad Imran Khan; investigation, Mahmood Hassan Dalhat; resources, Hani Choudhry and Mohammad Imran Khan; data curation, Mahmood Hassan Dalhat, Mohammed Razeeth Shait Mohammed., Hind Ali Alkhatabi, and Mohammad Imran Khan; writing—original draft preparation, Mahmood Hassan Dalhat; writing—review and editing, Hani Choudhry and Mohammad Imran Khan; visualisation, Mahmood Hassan Dalhat supervision, Hani Choudhry and Mohammad Imran Khan. All authors have read and agreed to the published version of the paper.

## CONFLICT OF INTEREST

The authors declare that there is no conflict of interest that could be perceived as prejudicing the impartiality of the research reported.

## Supporting information

Supporting Information

Table S1

Table S2

Table S3

Table S4

Table S5

## Data Availability

The authors declare that the main data for the findings in the study are available within the article and supporting information file. The RNA‐seq and acRIP‐seq data generated for this study were deposited at NCBI GEO DataSets with accession numbers GSE210086 and GSE210017.
