# Supplementary material for: NAT10: An RNA cytidine transferase regulates fatty acid metabolism in cancer cells
Source: Clin Transl Med. 2022 Sep 23;12(9):e1045. doi: 10.1002/ctm2.1045 (PMC9505754; doi:10.1002/ctm2.1045)
Supplement: Supplementary file 1 — Supporting Information [file CTM2-12-e1045-s004.docx]

Supporting Information

**N****AT10, an RNA cytidine transferase regulates fatty acid metabolism in cancer cells**

*Mahmood Hassan Dalhat^1,2^,* *Mohammed Razeeth Shait Mohammed^1,2^, Hind Ali Alkhatabi^3^, Mohd Rehan^4^,^5^, Aamir Ahmad^6^, Hani Choudhry^1,2^, Mohammad Imran Khan^1,2*^*

^1^ Department of Biochemistry, King Abdulaziz University, Jeddah 21589, Saudi Arabia

^2^ Centre for Artificial Intelligence in Precision Medicines, King Abdulaziz University, Jeddah 21589, Saudi Arabia

^3^ Department of Biochemistry, College of Science, University of Jeddah, Jeddah 21589, Saudi Arabia

^4^ King Fahd Medical Research Centre, King Abdulaziz University. P. O Box 80216, Jeddah 21589, Saudi Arabia

^5^ Department of Medical Laboratory Technology, Faculty of Applied Medical Sciences, King Abdulaziz University. P. O Box 80216, Jeddah 21589, Saudi Arabia

^6^ Translational Research Institute, Hamad Medical Corporation, Doha 3050, Qatar

*Correspondence: [mikhan@kau.edu.sa](mailto:mikhan@kau.edu.sa)


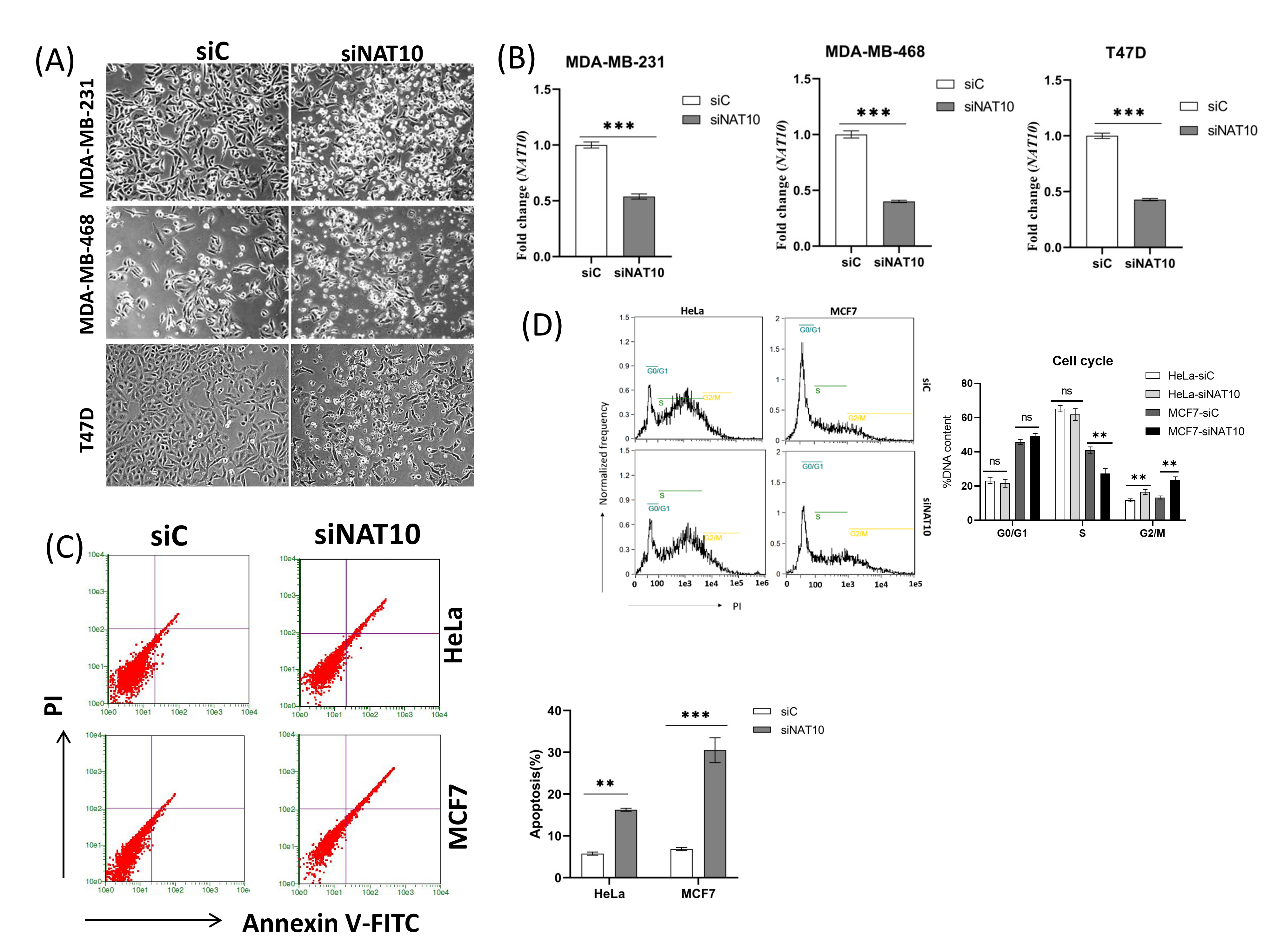


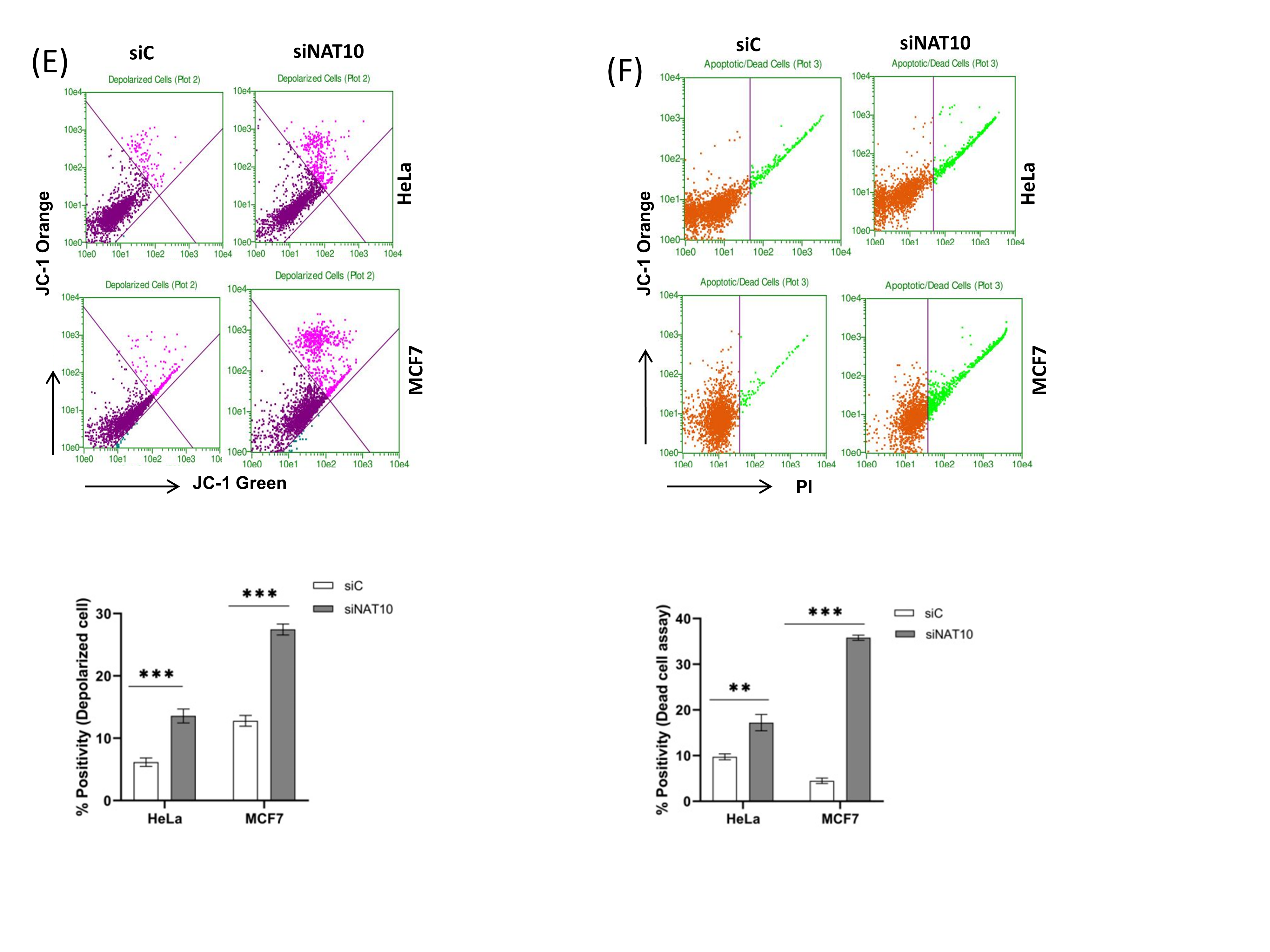


Figure S1. Figure S1. (A) Morphology of breast cancer cells post transfection with NAT10 siRNA. (B) Gene expression of NAT10 in breast cancer cells post transfection with NAT10 siRNA. (C) Apoptosis assay of HeLa and MCF7 in NAT10 transfected cells. (D) Cell cycle assay of HeLa and MCF7 cells in NAT10 transfected cells. Data in Figs C and D are represented as mean ± SEM (n=3) and p-value is calculated using student ttest. ***p<.001; **p<.01; and ns>.05. (E) Mitochondrial membrane potential of HeLa and MCF7 transfected with NAT10 siRNA. (F) Dead cell assay of HeLa and MCF7 transfected with NAT10 siRNA. Data in Figs. E and F are represented as mean ± SEM (n=3) and p-value is calculated using 2-way ANOVA, Sidak’s multiple comparison test.**p<.01 and ***p<.001.


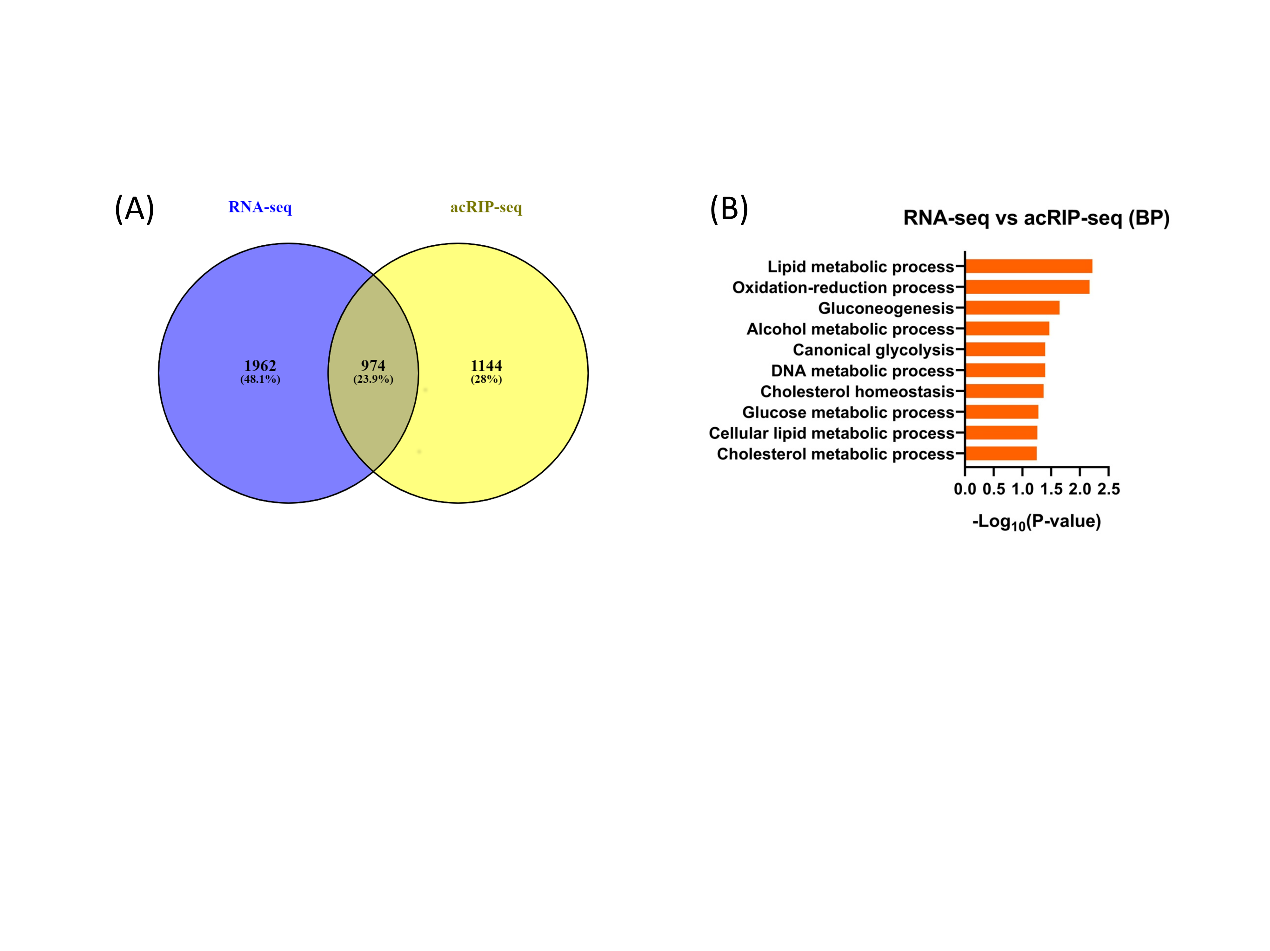


Figure S2. (A) Venn diagram of RNA-seq differentially down-regulated genes vs acRIP-seq retrieved from analysis of data from public database involving NAT10 knockout HeLa cells (GSE102113). (B) Biological process of the overlapping genes of RNA-seq differentially down-regulated genes vs acRIP-seq (974) using the functional annotation webserver DAVID.


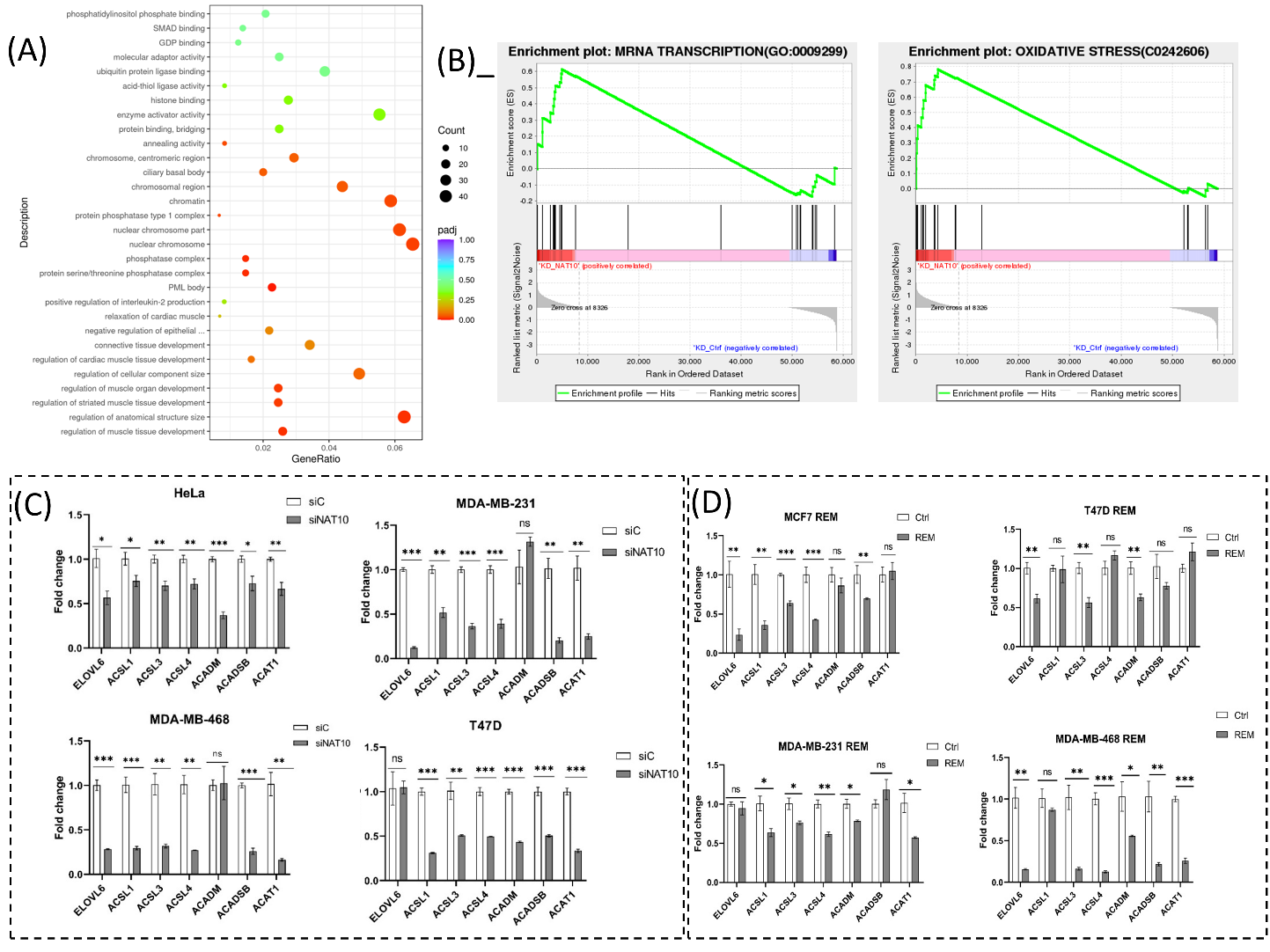


Figure S3. (A) Biological process of obtained from gene ontology of differentially downregulated genes in MCF7 siNAT10 knockdown cells. (B) GSEA enrichment plot of top enrichment phenotypes post knockdown with NAT10 siRNA. (C) Expression of identified fatty acid metabolic genes validation in NAT10 depleted HeLa and other breast cancer cells; MDA-MB-231, MDA-MB-468, and T47D. (D) Expression of identified fatty acid metabolic genes in Remodelin treated MCF7 and other breast cancer cells; MDA-MB-231, MDA-MB-468, and T47D. Bars are presented as mean ± SEM (n=3) and statistical significance is calculated using student 2-tailed t-test .*p<.05, **p<.01, ***p<.001 and ns>.05


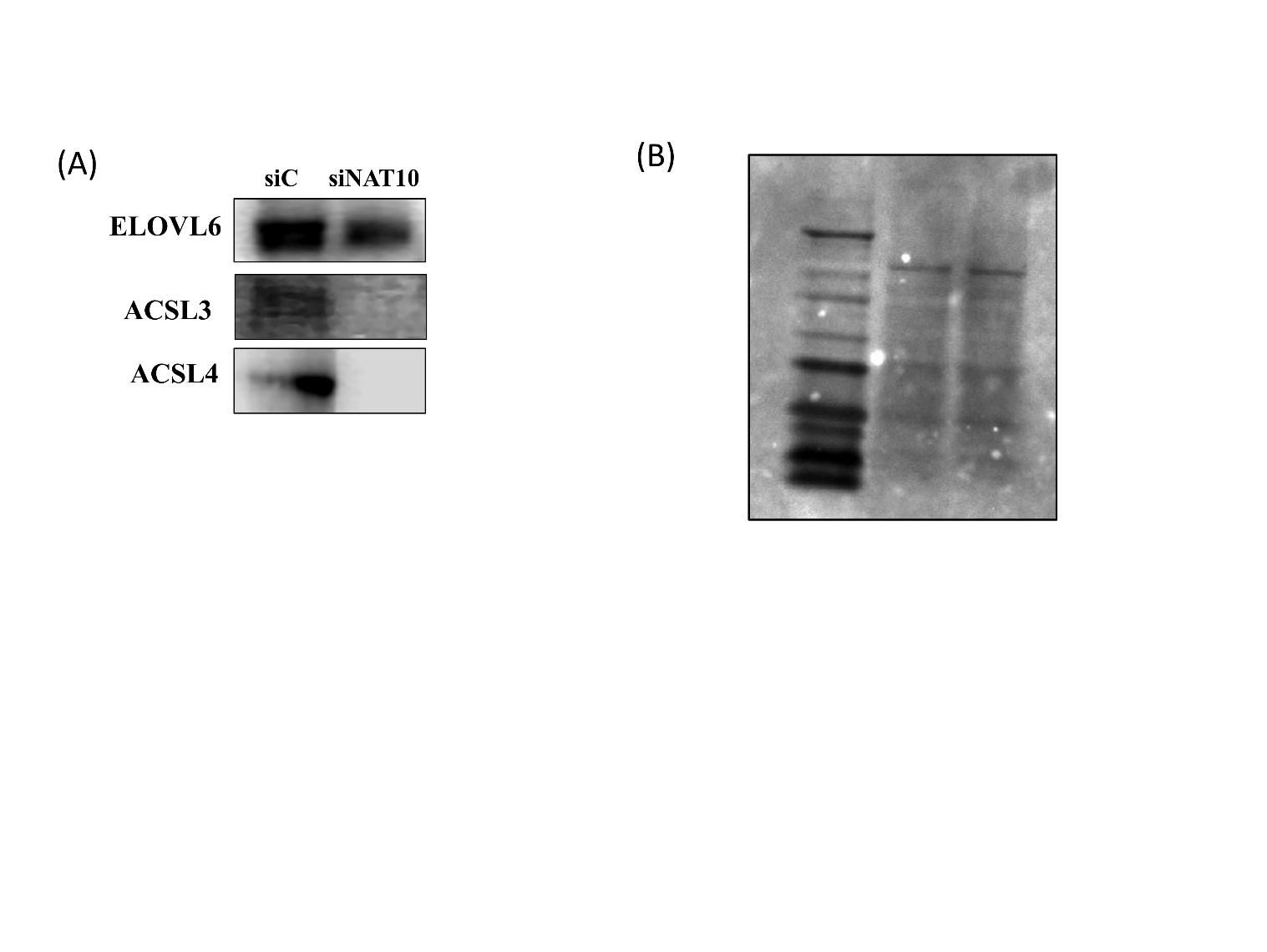


Figure S4. (A) Protein expression of ELOVL6, ACSL1, ACSL3, and ACSL4 in MCF7 transfected with NAT10 siRNA. (B) Ponceau stain image of MCF7transfected with NAT10 siRNA.


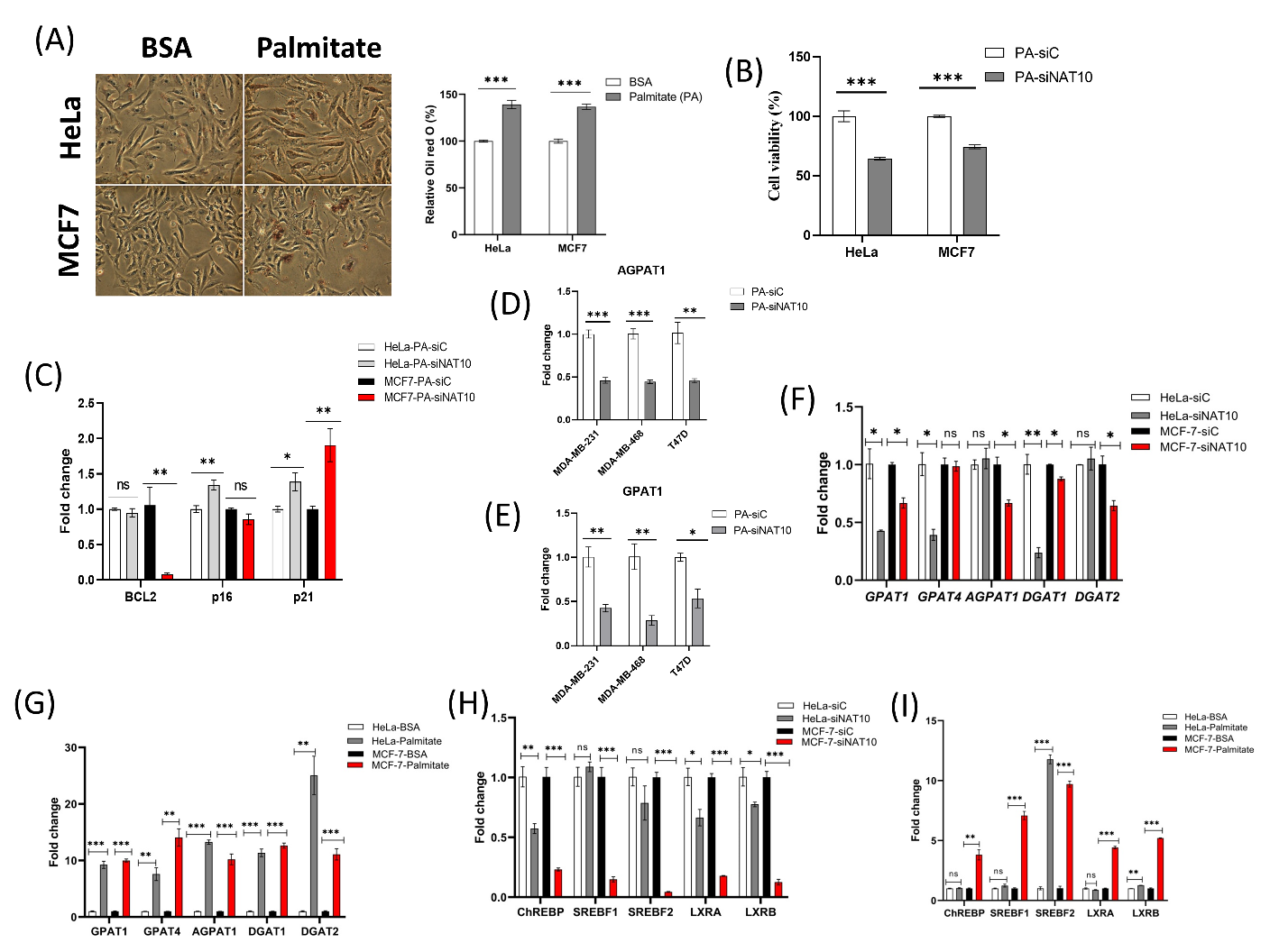


Figure S5. (A) Oil red O staining of palmitate loaded HeLa and MCF7 (baseline). (B) Cell viability assay of palmitate loaded cancer cells transfected with NAT10 siRNA. C) Expression of cell survival genes in palmitate loaded cancer cells transfected with NAT10 siRNA. (D-E) Expression levels of AGPAT1 and GPAT1 in palmitate loaded cancer cells transfected with NAT10 siRNA breast cancer cells. (F-G) Expression of genes associated with triglycerides biosynthesis in NAT10 knockdown and palmitate administered HeLa and MCF7. (H-I) Expression of lipogenic transcription factor genes in NAT10 knockdown and palmitate administered HeLa and MCF7. Data are presented as mean ± SEM (n=3) and statistical significance is calculated using student 2-tailed t-test.*p<.05, **p<.01, ***p<.001 and ns>.05.
